# Combined oral contraceptive pill compared with no medical treatment in the management of polycystic ovary syndrome: A systematic review

**DOI:** 10.1111/cen.14913

**Published:** 2023-03-27

**Authors:** Maria Forslund, Johanna Melin, Simon Alesi, Terhi Piltonen, Daniela Romualdi, Chau Thien Tay, Selma Witchel, Alexia Pena, Aya Mousa, Helena Teede

**Affiliations:** ^1^ Department of Obstetrics and Gynecology, Institute of Clinical Sciences, Sahlgrenska Academy University of Gothenburg Gothenburg Sweden; ^2^ Monash Centre for Health Research & Implementation, School of Public Health and Preventive Medicine, Faculty of Medicine, Nursing and Health Sciences Monash University Melbourne Victori Australia; ^3^ Department of Obstetrics and Gynecology, Helsinki University Hospital University of Helsinki Helsinki Finland; ^4^ Department of Obstetrics and Gynecology, Research Unit of Clinical Medicine and Medical research Centre, Oulu University Hospital University of Oulu Oulu Finland; ^5^ Department of Obstetrics and Gynecology Fondazione Policlinico Universitario Agostino Gemelli IRCCS Italy; ^6^ Department of Pediatrics, Division of Pediatric Endocrinology UPMC Children's Hospital of Pittsburgh Pittsburgh Pennsylvania USA; ^7^ Discipline of Paedriatics The University of Adelaide and Robinson Research Institute North Adelaide Australia; ^8^ Department of Diabetes and Vascular Medicine Monash Health Melbourne Victoria Australia

**Keywords:** combined oral contraceptive pills, hyperandrogenism, irregular cycles, polycystic ovary syndrome, treatment

## Abstract

**Objective:**

As part of the update of the International Evidence‐Based Guidelines for the Assessment and Management of polycystic ovary syndrome (PCOS), a systematic review was performed to inform evidence‐based recommendations.

**Design:**

Systematic review. Only randomised controlled trial were included.

**Patients:**

Women with PCOS; the use of combined oral contraceptive pills (COCP) was compared with no medical treatment.

**Measurements:**

Outcomes were designed in collaboration with clinical experts, researchers, and consumers. Critical outcomes included hirsutism, irregular cycles, quality of life, body mass index (BMI), and weight.

**Results:**

1660 publications were identified, but only four studies were included. No studies could be combined for meta‐analysis. COCP treatment improved cycle regularity compared with no medical treatment (100% vs. 0%, with low certainty of evidence). COCP showed no difference in improvement of hirsutism or BMI compared with placebo or lifestyle; a lower weight after COCP compared with no treatment (mean difference [MD] −8.0 (95% confidence interval, CI −11.67); −4.33 kg); and improvement in quality of life (MD 1.2 [95% CI 0.96]; 1.44), but these results were all very low certainty of evidence.

**Conclusion:**

Results show that COCP benefit cycle regulation, but other benefits or potential adverse effects were only identified with very low certainty of evidence. The COCP is frontline medical treatment in PCOS, but this is still based on established efficacy in the broader general population. Our results show that research in PCOS is seriously lacking and should be prioritised to capture core reproductive, metabolic and psychological outcomes important in PCOS.

## 1 INTRODUCTION

Polycystic ovary syndrome (PCOS) is a common condition, affecting 8%–13% of women.^1^ PCOS is characterised by complex genetic architecture with genes clustering around both reproductive and metabolic factors.^2^ Clinical diagnosis is based on modified Rotterdam criteria, requiring two out of three of the following: clinical and or biochemical hyperandrogenism, oligo/anovulation with irregular menstrual cycles and polycystic ovarian morphology (PCOM) on ultrasound, in adults. In adolescents, both hyperandrogenism and ovulatory disturbance are required as PCOM is a nonspecific finding among adolescent girls.^3^ PCOS is also a metabolic disorder with features including obesity, insulin resistance, pregnancy complications, hypertension, dyslipidemia, impaired glucose tolerance, diabetes, and cardiovascular disease.^3^ Psychological aspects are also prominent and include anxiety, depression, and eating disorders.^4^

Depending on the clinical manifestations, different treatments can be used. Primary treatment involves multicomponent lifestyle interventions, which should be recommended to all women with PCOS.^3^ Combined oral contraceptive pills (COCP) is one of the most frequently used medical treatments in PCOS to ameliorate hyperandrogenism and manage irregular menstrual cycles.^3^ A COCP contains both an oestrogen and a progestogen and is approved for contraceptive use. Most COCPs contain ethinyl estradiol (EE) as the oestrogen compound, however, during the recent years, products containing more natural‐like oestrogens have also been introduced. Several different progestogens are available, across different generations with variable properties bringing potentially distinguishable side‐effect profiles similar to different oestrogen components.^5^, ^6^

COCP provides hormone replacement therapy where the hypothalamus–pituitary axis is effectively down regulated by oestrogen/progestogen and thereby enabling regulation of menstrual cycles. When the COCP is ceased (usually for 4–7 days per month when not used continuously) a withdrawal bleed is induced, resulting in a regular monthly bleeding pattern. COCPs are key contraceptive agents, important for women with PCOS not currently seeking pregnancy. Even though women with PCOS can experience difficulties conceiving spontaneously, effective contraception is essential when a pregnancy is not desired.^7^

COCP treatment can be administered cyclically or continuously. Regardless, cyclic or continuous administration protects the endometrium from unopposed oestrogen in women with PCOS who do not ovulate as regularly and often lack adequate endometrial progesterone exposure. The longstanding unopposed oestrogen stimulation is the main hypothesis behind the potentially increased risk for endometrial cancer in women with PCOS.^8^ From large studies in the general populations, it is well‐known that COCP use is an effective long‐term protection against endometrial cancer.^9^

Importantly, COCP improves hyperandrogenism via different mechanisms, primarily by stimulation of sex hormone binding globulin (SHBG) production, the major binding protein for testosterone, thereby decreasing free circulating androgens.^10^ COCP also decreases luteinizing hormone secretion, which leads to decreased ovarian androgen production.^11^, ^12^ In addition, some progestins has antiandrogenic properties via competition with endogenous androgens at androgen receptors (e.g., in skin and hair) and by inhibition of the 5‐α‐reductase type I enzyme expressed in skin.^13^

In PCOS, COCP thus addresses two of the main symptoms in PCOS, namely irregular cycles and clinical signs of hyperandrogenism. These symptoms have also been shown to be associated with decreased health‐related quality of life (HRQoL) in women with PCOS.^14^ Here, we aimed to explore the effects of COCP compared with no treatment, placebo or lifestyle intervention in PCOS as a common reproductive, metabolic, psychological condition. We sought to capture the best available evidence in the treatment of women with PCOS to guide the 2023 International Guideline Update and inform future research priorities.^3^, ^15^ A number of outcomes were identified as important and were included in this systematic review, those identified a priori as critically important by clinical experts, researchers, and consumers were hirsutism, irregular cycles, quality of life, body mass index (BMI) and weight.

## 2 MATERIALS AND METHODS

This systematic review was conducted following the Preferred Reporting Items for Systematic Reviews and Meta‐Analysis (PRISMA) guidelines^16^ as part of the evidence synthesis for the update of the International Evidence‐Based Guidelines for the Assessment and Management of PCOS. Key clinically relevant questions in PCOS care, details of the Patient, Intervention, Comparison and Outcomes (PICO) framework and prioritised outcomes were established through a Delphi process, involving 700 clinicians, academic opinion leaders, and consumers.^16^ The PCOS guidelines evidence team (A. M., C. T. T.), consumers and leading experts across the world (including H. T., T. P., A. P., D. R., S. W.) support the 2023 Guideline Update. The protocol for the systematic review was registered in Prospero (CRD42022345640) on July 23, 2022, before full‐text screening. In this publication, results regarding COCP versus no medical treatment (controls/placebo/lifestyle treatment) are reported.

### 2.1 Search strategy

*The search was done as part of the PCOS guideline update and included three medical treatments (COCP, metformin and anti‐androgens)*. The search for studies published until 2017 had been done for the previous international guidelines and thus captured there.^17^ All included and excluded studies from that search were re‐evaluated here. The search was updated on July 8, 2022, using the same search string, but limited to publications from 2017 and forward. The search was conducted using the following databases: Medline, EMBASE, All EBM, CINAHL, and PsychInfo, and was thus again performed for three medical treatments (COCP, metformin, and antiandrogens) as part of the evidence for the updated guidelines. The relevant overall question for COCP was: “Is the oral contraceptive pill alone or in combination effective for management of hormonal and clinical PCOS features in adolescents and adults with PCOS?”

This publication is limited to comparisons between COCP compared with controls, placebo and/or lifestyle treatment. Supporting Information: Table 1 presents the search strategy in detail.

### 2.2 Selection criteria

The PICO was defined with input from women and experts as noted above.

The *population* relevant for this systematic review were females with PCOS diagnosed by Rotterdam, National Institute of Health (NIH) or Androgen Excess Society (AES) (now Androgen Excess and PCOS [AE‐PCOS] Society) criteria. Exclusion criteria were women less than 2 years postmenarche, and women with important comorbidities including type 2 diabetes and major depression.

*Interventions* included all types of COCP with a treatment duration of at least 3 months, however, to assess the outcome hirsutism, a minimum of 6 months treatment was required. *Comparisons* were no medical treatment, including controls (no treatment), placebo or lifestyle intervention.

The *outcomes* that a priori had been prioritised^3^, ^16^, ^18^ were clinical androgenicity (hirsutism as indicated by Ferriman Gallwey [FG] score), biochemical androgenicity (free androgen index [FAI], testosterone, SHBG, dehydroepiandrosterone sulphate [DHEAS], androstenedione), irregular cycles, metabolic outcomes (Homoeostatic Model Assessment for Insulin Resistance [HOMA‐IR], clamptest, oral glucose tolerance test [OGTT], cholesterol, low density lipoprotein (LDL), high density lipoprotein (HDL), triglycerides, C‐reactive protein [CRP]), psychological outcomes (HRQoL, depression), anthropometric outcomes (weight, BMI, waist‐hip ratio), thromboembolic events, plasminogen activator inhibitor (PAI)‐1 levels and adverse effects. The outcome was included in the systematic review regardless of effect estimate.

Only randomised controlled trials (RCTs) were included.

Studies were selected and appraised by reviewers (M. F., J. M., S. A.), using study selection and appraisal criteria established a priori, in the tool Covidence (www.covidence.org). The articles were first reviewed by title and abstract by two reviewers. If no decision could be reached based on title and abstract alone, the full text was also retrieved. Only studies that were unsuitable for the PICO for any of the three medical treatments were excluded. Full‐text screening was done by two reviewers (M. F., J. M., S. A.). Conflicts were resolved by discussion. In addition to the studies included in previous guidelines, the excluded list from that search was reviewed, and studies were included if they met PICO criteria, for comprehensiveness. Systematic reviews and evidence‐based guidelines were not included but screened manually for additional references.

### 2.3 Data extraction and quality assessment

Data extraction was done following a predefined structure by one author (M. F.) and checked by an additional author (J. M.). To evaluate the risk of bias (RoB) at individual study level, an adapted version of RoB2 was used,^19^ and in addition any conflicts of interest found were assessed. Two authors (M. F. and J. M./S. A.) independently assessed the RoB of each article. If disagreements arose, conflicts were solved by discussion. The assessments were conducted in the tool Covidence.

To estimate the strength of evidence at the outcome level, the GRADE (Grading of Recommendations, Assessment, Development and Evaluations) approach was used.^20^ One GRADE‐assessment was conducted for each outcome by one author (M. F.) and double‐checked by another author (S. A./J. M).

### 2.4 Statistical analysis

All biochemical outcomes were converted to SI units when appropriate. When standard errors (SE) were reported, this was recalculated to standard deviation (SD) using conventional methods. *p* Values were, if possible, calculated in Review Manager version 5.4.1. when not reported in the publications.

## 3 RESULTS

### 3.1 Study selection

**Figure 1:**
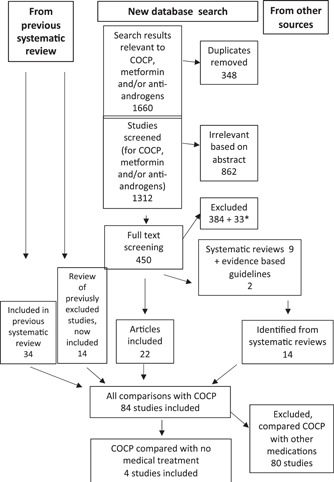
PRISMA flowchart. The search was performed for the update of the international evidence‐based guidelines for the assessment and management of PCOS, and covered three questions (treatment with COCP, metformin and antiandrogens). In this systematic review, results comparing COCP with no medical treatment is included. *384 studies were excluded; an additional 33 studies were included in metformin and/or antiandrogen questions, but not in COCP. COCP, combined oral contraceptive pills; PCOS,polycystic ovary syndrome; PRISMA, Preferred Reporting Items for Systematic Reviews and Meta‐Analysis.

Three different comparisons were targeted: COCP versus controls, COCP versus placebo and COCP versus lifestyle. A flowchart of included/excluded studies is shown in Figure 1. Reasons for exclusion are shown in Supporting Information: Table 2.

### 3.2 Characteristics of included studies

**Figure 2:**
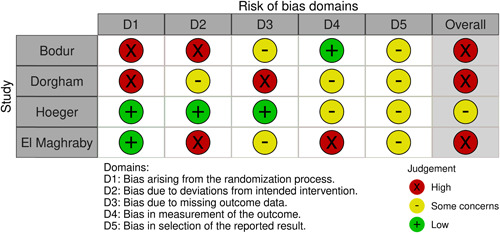
Risk of bias assessments of the included studies. [Color figure can be viewed at wileyonlinelibrary.com]

Four RCTs were identified for these three comparisons, with a study duration between 6 and 24 months. These studies had been published between 2008 and 2021. Countries of origin were Turkey (*n* = 1),^21^ Egypt (*n* = 2),^22^, ^23^ and the United States (*n* = 1).^24^ Characteristics of included studies are shown in Table 1. Two of the RCTs included adults with PCOS and two were restricted to adolescents. Three of the studies had a high RoB and one had moderate risk. RoB assessments are shown in Figure 2.

**Table 1:** Included studies in comparison COCP versus no medical treatment.

References	RoB	Comparisons	Country, duration	*N*	Mean age	Mean BMI	PCOS diagnosis	Menarche age	Smokers	Comments	Outcomes relevant to this review
Bodur et al.^21^	High	1: 30 μg EE + 3 mg DRSP 2: 1700 g metformin 3: 30 μg EE + 3 mg DRSP + 1700 g metformin 4: controls, no mediciation	Turkey 6 months	1:17 2:17 3:12 4:15	1: 26.62 ± 4.92 2: 26.24 ± 3.96 3: 27.35 ± 5.65 4: 29.18 ± 5.20	1: 23.45 ± 3.40 2: 25.06 ± 3.08 3: 25.11 ± 3.75 4: 23.82 ± 2.80	Rott	NR	NR		CRP, PAI‐1, glucose, HOMA
Dorgham et al.^23^	High	1: only laser (controls) 2: laser + metformin 500 mg 3: laser 35 μg EE + 2 mg cyproteron acetate	Egypt 6 months (results available also for 3 months)	1:50 2:50 3:50	NR	NR	Rott	NR	NR	All received laser! Facial hirsutism required	HRQoL (VAS, DLQI, HLQI)
El Maghraby et al.^22^	High	1: 30ug EE + 15 mg progestin/day 2: 1700 mg MET/day 3: controls, no medication	Egypt (6, 12, 18, 24 m) 2 years	1:33 2:32 3:25	1: 16.90 ± 1.60 2: 17.20 ± 2.00 3: 17.00 ± 2.10	NR	Rott	NR	NR	adolescents	total testosterone, insulin, weight, improvement in cycle regularity, OGTT
Hoeger et al.^24^	Mod	1. 30 μg EE + 0.15 mg DSG 2. Placebo 3. Lifestyle addressing diet, exercise, behaviour 4: 1700 mg met/day	United States 6 months	1:10 2:10 3:8 4:6	1: 15.4±1.4 2: 15.4±1.7 3: 15.4±1.2 4: 15.4±1.7	1: 37.8 ± 5.1 2: 36.1 ± 7.5 3: 37.8 ± 8.2 4: 36.1 ± 7.5	Rott	NR	All nonsmokers	Obese cohort Adolescents	BMI, FG score, FAI, total testosterone, SHBG, chol, LDL, HDL, TG, CRP, BMI, PAI‐1

Abbreviations: BMI, body mass index; COCP, combined oral contraceptive pills; CRP, C‐reactive protein; DLQI, Dermatology LifeQuality Index; DRSP, dropserinone; DSG, desogestrel; EE, ethinyl estradiol; FAI, free androgen index; FG, Ferriman Gallwey; HDL, density lipoprotein; HLQI, Hisutism Life Quality Index; HOMA, Homoeostatic Model Assessment; HRQoL, health‐related quality of life; LDL, low density lipoprotein; NR, not reported; OGTT, oral glucose tolerance test; PAI, plasminogen activator inhibitor; PCOS, polycystic ovary syndrome; RoB, risk of bias; SHBG, sex hormone binding globulin.

El Maghraby et al.^22^ included adolescent girls, 15–20 years, presenting with hirsutism, acne and menstrual disturbances. PCOS was defined as a combination of oligomenorrhea and biochemical hyperandrogenism, mean BMI or age at menarche was not reported. Treatment with COCP (30 μg EE + 15 mg progestogen, type not reported), *n* = 40, was compared with no treatment (control group), *n* = 39. The study duration was 24 months, with 18% dropout rate in the COCP group and 36% dropout rate in the control group. The study was not blinded, baseline characteristics were only shown for a few outcomes, and the dropout rate was not the same in the two groups, thus the study was assessed as high RoB.

Bodur et al.^21^ included nonobese (BMI < 30 kg/m^2^) women, aged 18–39 years, with PCOS diagnosed through Rotterdam criteria. Treatment with 30 µg EE + 3 mg drospirenone (*n* = 21) for 6 months was compared with a control group (*n* = 17). The dropout rate was 19% versus 13% for COCP and controls, respectively. The study was not blinded, there was no comparison of important PCOS manifestations such as hyperandrogenism at baseline was presented, and there was no registered study protocol, hence the study was assessed as high RoB.

In a study by Dorgham et al.,^23^ all groups, including the control group received laser treatment, and in this study, facial hirsutism was an additional inclusion criterion. Age and BMI were not reported. Treatment for 6 months with COCP (35 μg EE + 2 mg cyproterone acetate, *n* = 50) was compared with controls (*n* = 50). HRQoL in the areas of hirsutism and dermatology were assessed. The study had a high RoB in the absence of blinding, lack of baseline data and no report regarding study dropouts.

Hoeger et al.^24^ had multiple arms and included adolescents with a mean age of 15 years, time since menarche not reported. The study compared COCP (30 µg EE + 0.15 mg desogestrel, *n* = 11) with placebo (*n* = 11) with one dropout per arm, and mean BMI was 38 versus 36 kg/m^2^, respectively. The study also included one lifestyle arm (*n* = 11, three dropouts during the 24 weeks), with a baseline BMI of 36 kg/m^2^. This study had a moderate RoB, largely because outcome assessors were not blinded and potential conflict of interest.

Thus, three studies^21^, ^22^, ^23^ compared COCP with a no‐treatment control group and results, including reported adverse events are shown in Table 2. One study^24^ compared COCP with placebo, and the same study^24^ also compared COCP with lifestyle, these results are shown in Tables 3 and 4. Results are summarised here according to outcome group, and reasons for GRADE assessments of the different outcomes are given in Table 2, 3, 4. Overall, evidence were mostly of very low certainty, except for menstrual cycles, and thus effect estimates are not reported here, with the exception of the outcomes a priori defined as critically important. Results are shown in Tables 2, 3, 4.

**Table 2:** Comparison between treatment with COCP and no‐treatment controls.

Outcome	References	Time point (month)	*N*	COCP mean ± SD	No‐treatment controls mean ± SD	*p* Value	Favours	Certainty
Weight (kg)	El Maghraby et al.^22^	24	COCP 33 Controls 25	91.00 ± 3.00	99.00 ± 9.00	<.0001	COCP	⊕◯◯◯ Very low
Testosterone (μg/mL)	El Maghraby et al.^22^	24	COCP 33 Controls 25	0.70 ± 0.20	1.50 ± 0.40	<.0001	COCP	⊕◯◯◯ Very low
Insulin (pmol/L)	El Maghraby et al.^22^	24	COCP 33 Controls 25	131.9 ± 27.8	152.8 ± 20.8	<.01	COCP	⊕◯◯◯ Very low
Glucose (mmol/L)	Bodur et al.^21^	6	COCP 17 Controls 15	4.57 ± 0.48	4.59 ± 0.25	.85	No difference	⊕◯◯◯ Very low
OGTT (after‐load insulin)	El Maghraby et al.^22^	24	COCP 33 Controls 25	187 ± 22	111 ± 12	<.0001	No‐treatment controls	⊕◯◯◯ Very low
HOMA‐IR	Bodur et al.^21^	6	OCP 17 Controls 15	3.10 ± 2.01	2.20 ± 0.59	.11	No difference	⊕◯◯◯ Very low
CRP (mg/L)	Bodur et al.^21^	6	COCP 17 Controls 15	0.87 ± 0.20	0.59 ± 0.36	<.01	No‐treatment controls	⊕◯◯◯ Very low
PAI‐1 (ng/mL)	Bodur et al.^21^	6	OCP 17 Controls 15	19.19 ± 2.97	14.59 ± 2.50	<.0001	No‐treatment controls	⊕◯◯◯ Very low
HRQoL VAS (scale 0‐10)	Dorgham et al.^23^	6	COCP 50 Controls 50	4.2 ± 0.6	3.0 ± 0.6	<.0001	COCP	⊕◯◯◯ Very low
HRQoL DLQI	Dorgham et al.^23^	6	COCP 50 Controls 50	1.0 ± 0.6	5.5 ± 2.5	.002	COCP	⊕◯◯◯ Very low
HRQoL HLQI 0–22, none to severe problems	Dorgham et al.^23^	6	COCP 50 Controls 50	1.45 ± 0.5	6.5 ± 2.3	.002	COCP	⊕◯◯◯ Very low
Improvement in cycle regularity	El Maghraby et al.^22^	NR	*N* (%)	40/40 (100%)	0/40 (0%)	NA	COCP	⊕⊕◯◯ Low

	Adverse effects				
Weight gain	El Maghraby et al.^22^			7/40	0/39
Nausea	Bodur et al.^21^			1/21	0/17
Dizziness	Bodur et al.^21^			0/21	0/17
Sexual reluctance	Bodur et al.^21^			1/21	0/17
Worsening of symptoms	El Maghraby et al.^22^			0/40	7/39
Other	El Maghraby et al.^22^			2/40	0/39
	Bodur et al.^21^			Totally 4/21 dropouts, as above + pregnancy, hirsutism	2/17 dropouts, unknown reason

*Note*: All results had a very low certainty of evidence due to very serious risk of bias, and very serious imprecision. The outcome improvement of cycle regularity was upgraded once for large effect.

Abbreviations: COCP, combined oral contraceptive pills; CRP, C‐reactive protein; DLQI, Dermatology LifeQuality Index; HLQI, Hisutism Life Quality Index; HOMA‐IR, Homoeostasis Model Assessment for Insulin Resistance; HRQoL, health‐related quality of life; NA, not applicable; NR, not reported; OGTT, Oral glucose Tolerance Test; PAI, plasminogen activator inhibitor.

^a^ El Maghraby et al.^22^ reports TT levels corresponding to extreme values and is thus not converted to SI units.

**Table 3:** Comparison between treatment with COCP and placebo, adapted from Hoeger et al.^24^

Outcome	Time point (month)	*N*	COCP mean ± SD	Placebo mean ± SD	*p* Value	Favours
BMI (kg/m^2^)	6	COCP 10 P 10	36.4 ± 5.4	35.5 ± 6.8	.74	No difference
Hirsutism (FG score)	6	COCP 10 P 10	8.6 ± 2.1	11.6 ± 4.9	.09	No difference
FAI	6	COCP 10 P 10	2.4 ± 2.5	16.8 ± 11.2	<.001	COCP
Testosterone (nmol/L)	6	COCP 10 P 10	1.20 ± 0.99	2.48 ± 1.17	.02	COCP
SHBG (nmol/L)	6	COCP 10 P 10	93.2 ± 66.5	19.1 ± 9.4	<.01	Higher after COCP
Insulin (pmol/L)	6	C 10 P 10	143.7 ± 73.6	202.1 ± 170.1	.33	No difference
Glucose (mmol/L)	6	C 10 P 10	4.60 ± 0.54	4.80 ± 0.30	.31	No difference
Cholesterol (mmol/L)	6	COCP 10 P 10	4.88 ± 0.54	4.07 ± 1.38	.10	No difference
LDL (mmol/L)	6	COCP 10 P 10	3.33 ± 0.97	2.95 ± 0.70	.33	No difference
HDL (mmol/L)	6	COCP 10 P 10	1.23 ± 0.26	1.13 ± 0.23	.35	No difference
TG (mmol/L)	6	COCP 10 P 10	1.09 ± 0.46	0.98 ± 0.28	.56	No difference
CRP (mg/L)	6	C 10 P 10	9.5 ± 7.4	4.2 ± 2.8	<.05	Placebo
PAI‐1 (ng/mL)	6	C 10 P 10	29.5 ± 20.6	48.0 ± 45.9	.26	No difference

	Adverse effects			
Gastrointestinal problems	6	*N* (%)	0/11 (0%)	1/11 (9%)

*Note*: All results had a very low certainty of evidence due to serious risk of bias, and very serious imprecision.

Abbreviations: BMI, body mass index; COCP, combined oral contraceptive pills; CRP, C‐reactive protein; FAI, free androgen index; FG, Ferriman Gallwey; HDL, high density lipoprotein; LDL, low density lipoprotein; P, placebo; PAI, plasminogen activator inhibitor; SHBG, sex hormone binding globulin.

**Table 4:** Comparison between treatment with COCP and lifestyle, adapted from Hoeger et al.^24^

Outcome	Time point	*N*	COCP mean ± SD	Lifestyle mean ± SD	*p* Value	Favours
BMI (Kg/m^2^)	6 m	COCP 10 LS 8	36.4 ± 5.4	34.9 ± 7.0	.61	No difference
Hirsutism (FG score)	6 m	COCP 10 LS 8	8.6 ± 2.1	8.2 ± 2.0	ND	No difference
FAI	6 m	COCP 10 LS 8	2.4 ± 2.5	9.5 ± 5.3	ND	No difference
Testosterone (nmol/L)	6 m	COCP 10 LS 8	1.20 ± 0.99	2.24 ± 1.05	<.05	COCP
SHBG (nmol/L)	6 m	COCP 10 LS 8	93.2 ± 66.5	32.0 ± 21.7	ND	No difference
Insulin (pmol/L)	6 m	COCP 10 LS 8	143.7 ± 73.6	152.8 ± 72.9	.80	No difference
Glucose (mmol/L)	6 m	COCP 10 LS 8	4.60 ± 0.54	4.54 ± 0.51	.83	No difference
Cholesterol (mmol/L)	6 m	COCP 10 LS 8	4.88 ± 0.54	4.05 ± 0.80	ND	No difference
LDL (mmol/L)	6 m	COCP 10 LS 8	3.33 ± 0.97	2.62 ± 0.84	<.05	Lifestyle
HDL (mmol/L)	6 m	COCP 10 LS 8	1.23 ± 0.26	1.05 ± 0.20	ND	No difference
TG (mmol/L)	6 m	COCP 10 LS 8	1.09 ± 0.46	0.98 ± 0.28	<.05	Lifestyle
CRP (mg/L)	6 m	COCP 10 LS 8	9.5 ± 7.4	3.8 ± 3.6	.06	No difference
PAI‐1 (ng/mL)	6 m	COCP 10 LS	29.5 ± 20.6	45.0 ± 25.6	.17	No difference

*Note*: All results had a very low certainty of evidence due to serious risk of bias, and very serious imprecision.

Abbreviations: BMI, body mass index; COCP, combined oral contraceptive pills; CRP, C‐reactive protein; FAI, free androgen index; FG, Ferriman Gallwey; HDL, high density lipoprotein; LDL, low density lipoprotein; LS, lifestyle; PAI, plasminogen activator inhibitor; SHBG, sex hormone binding globulin.

### 3.3 Anthropometry

Weight was lower after COCP treatment [mean difference, MD −8.0 (95% confidence interval, CI −11.67; −4.33 kg)] compared with no‐treatment controls, with very low certainty of evidence. BMI did not differ between COCP and placebo or between COCP and lifestyle, with very low certainty of evidence.

### 3.4 Menstrual cycles

In the comparison COCP versus no‐treatment controls, COCP treatment resulted in improved cycle regularity (100% vs. 0%) with low certainty of evidence. In other comparisons, this outcome was not assessed.

### 3.5 Clinical and biochemical hyperandrogenism

Hirsutism did not differ between COCP‐treated women and women treated with placebo or lifestyle, respectively, with very low certainty of evidence. Total testosterone levels were lower after COCP treatment compared with no‐treatment controls, placebo, and lifestyle, respectively, with very low certainty of evidence. SHBG was higher after COCP compared with placebo, but no difference was seen compared with lifestyle, with very low certainty. FAI was lower after COCP treatment compared with placebo, but did not differ when comparing COCP and lifestyle, with very low certainty of evidence.

### 3.6 Insulin and glucose

Insulin levels were lower after COCP treatment, compared with no‐treatment controls, with very low certainty of evidence, but the COCP group had higher after‐load insulin levels after an oral glucose tolerance test, also with very low certainty. HOMA‐IR did not differ between COCP and no‐treatment controls, with very low certainty. Insulin levels did not differ compared with placebo or lifestyle, with very low certainty. Glucose did not differ in any of the comparisons with very low certainty.

### 3.7 Metabolic lipids, PAI and CRP

Only Hoeger et al. examined lipid profile with COCP use compared with no medical treatment.^24^ Cholesterol, LDL, HDL and triglycerides did not differ between COCP and placebo, with very low certainty. LDL and triglycerides were higher after COCP treatment compared with lifestyle with very low certainty of evidence, whereas HDL and cholesterol did not differ.

Higher levels of CRP were seen after COCP treatment compared with no‐treatment controls, and compared with the placebo group, but not compared with lifestyle, with very low certainty of evidence. Levels of PAI‐1 were higher after COCP treatment compared with controls, but there was no difference in PAI‐1 levels when compared with lifestyle or placebo, with very low certainty.

### 3.8 HRQoL and psychological outcomes

COCP was superior to no‐treatment controls regarding HRQoL overall, measured by VAS [MD 1.2 (95% CI 0.96; 1.44)], as well as in a dermatologic HRQoL questionnaire and a hirsutism HRQoL questionnaire, with very low certainty of evidence.

### 3.9 Adverse effects

COCP treatment was associated with more minor adverse effects than no‐treatment controls. The reported adverse effects are shown in Table 2. Adverse effects were not reported for the other comparisons.

## 4 DISCUSSION

This systematic review summarizes the knowledge of COCP treatment in PCOS compared with no medical treatment. These results form part of the evidence included in the 2023 update of the International Evidence‐Based Guidelines on the assessment and treatment of PCOS.^3^ As expected, COCP improved cycle regularity compared with control; however, here with low certainty of evidence. Additional evidence regarding COCP treatment versus no medical treatment (controls/placebo/lifestyle) is currently only available at a very low certainty of evidence in women with PCOS.

### 4.1 Risks and benefits with COCP treatment

Abundant studies in the general female population over six decades, have provided unequivocal evidence on efficacy of COCP for contraception, cycle regulation and hyperandrogenism.^12^, ^25^ In a large meta‐analysis of healthy women, COCP decreased total testosterone, [MD, (95% CI) −0.49 nmol/L (−0.55; −0.42), increased SHBG levels, [MD (95% CI)] 99.08 nmol/L (86.43; 111.73), and decreased free testosterone, relative change 0.39 (0.35; 0.43), corresponding to a 61% decrease compared with baseline.^12^ However, COCP treatment is also associated with an increased risk of venous thrombosis (VTE) in the general population, with an estimated incidence of 1–6 per 10,000 women years in nonusers, compared with 8–10 per 10,000 women years in COCP users.^26^ Second‐generation progestogen COCPs are associated with less risk, even though the absolute differences are small.^26^ Elevated high‐sensitivity CRP levels are associated with an elevated risk of cardiovascular disease events, and CRP levels are often measured in studies as a metabolic risk marker.^27^ COCP, especially those containing EE, seem to increase thromboinflammatory proteins, including CRP levels,^28^, ^29^ although the significance of this finding is still unknown. However, interestingly, a recent national case–control study from Finland, including almost 600,000 women, found that the increased risk was associated with COCP‐containing EE, whereas no increased risk of VTE was seen with COCP‐containing estradiol combined with progestins other than cyproterone acetate.^6^ COCP use are also associated with an increased risk of arterial thrombosis, that is, myocardial infarction or ischaemic stroke, relative risk 1.6.^30^ There has also been a concern that COCP would have an unfavourable effect on glucose metabolism, but this has not been seen in healthy women.^28^, ^31^ In women with PCOS, results regarding effects on glucose metabolism are contradictory, potentially due to the diverse effects of the different progestins. Many patients are anxious about potential effects on weight, but this has not been shown in the general population.^32^ Nevertheless, an association between COCP use and mood alterations is suspected, despite limited evidence. In a Swedish cohort study, the use of both COCP and progestin‐only pills was associated with an increased risk of suicidal behaviour, with the greatest risk occurring 1 month after initiation of use (hazard ratio 1.7 and 2.8, respectively, after 1 month).^33^ In a Danish cohort study, COCP users had a higher risk of starting antidepressive treatment, compared with nonusers, relative risk 1.2,^34^ although a recent register study from Sweden was not able to confirm depression with COCP use.^35^

### 4.2 Evidence in women with PCOS

Women with PCOS differ from the general population since they are more hyperandrogenic and PCOS is associated with inherent metabolic disturbances including hyperinsulinemia and insulin resistance. Women with PCOS have higher BMI, are more prone to gain weight and also have an increased risk of psychiatric comorbidity.^36^, ^37^ In this systematic review involving women with PCOS compared with no medical treatment, most evidence, three studies, was found for the comparison COCP versus controls (no treatment). However, as the studies reported different outcomes, no meta‐analyses could be done. Regarding subjective outcomes, such as HRQoL, the use of a control group instead of placebo could possibly influence the results. Only one small study was found that compared COCP with placebo. In a systematic review and meta‐analysis from 2018, including both RCTs and non‐RCTs, Amiri and coworkers evaluated the effect of COCP treatment on hyperandrogenism in women with PCOS,^38^ 35 studies were included, of which 25 only had only one treatment group. They compared clinical and biochemical hyperandrogenism before and after COCP treatment and found improvement in both clinical and biochemical hyperandrogenism; however, no direct comparisons were done between treatments. Longer treatments (6–12 months) were suspected to be more effective in improving hirsutism compared to shorter durations (3 months), as MD compared with before treatment was higher for longer durations.^38^ Women with PCOS differ from the general population in having an increased prevalence and degree of hyperandrogenism, as well as having a different risk profile with an increased metabolic risk compared with the general population. To be able to compare benefits and risks of COCP treatment, studies comparing COCP with placebo are important, both for patients and health care workers, to be able to make the best possible treatment recommendations for the individual patient.

### 4.3 COCP versus lifestyle

Regarding the comparison COCP versus lifestyle, only one small study was identified in our review, with a total of 18 participants.^5^ However, another RCT by Legro et al., published in 2015,^39^ also compared COCP treatment with lifestyle intervention. However, in that study, participants randomised to the lifestyle arm were also given antiobesity medication if BMI > 30 kg/m^2^. As mean BMI was 35 kg/m^2^, the majority of patients were given antiobesity treatment, and thus, that RCT was not included in this systematic review. In addition, this study included only infertile women, and the COCP and lifestyle treatments were preconception interventions, after which the women underwent infertility treatment. Results from this RCT indicated improved subjective hirsutism after COCP treatment, as well as decreased testosterone and increased SHBG levels.^39^ No differences were evident between the groups regarding sexual function scores, but importantly, no difference was seen in sexual desire or sexual function after COCP treatment compared with before treatment.^40^

### 4.4 Research gaps

The majority of findings in this systematic review had a very low certainty of evidence. This is due to both serious imprecision given the lack of studies, with only one included study per outcome and comparison, as well as serious RoB given that three of the four studies included had a high RoB. There is a need for high‐quality RCTs addressing COCP treatment versus placebo with appropriate methodologies and statistical power. Misconceptions and misinformation persist across social media and other public platforms suggesting that women with PCOS do not need contraception due to decreased fertility/infertility. However, women with PCOS not actively seeking pregnancy should utilize an effective contraceptive method and obtain appropriate treatment for PCOS‐related symptoms. Myths around COCP use, such as weight gain, indicating that providing evidence‐based information on both COCP efficacy and side effects will benefit all. Importantly, studies regarding adverse effects have not been studied consistently, highlighting the need for high‐quality data.^18^ Having prespecified, and consistently examining, the most important adverse outcomes in future studies would increase available evidence for COCP treatment in PCOS, to enable patients to make informed decisions on proposed COCP treatment better.^18^

### 4.5 Strengths and weaknesses

The strengths of this systematic review include the well‐defined and clinically relevant question regarding COCP treatment in PCOS, with outcomes that have been defined and prioritised by multiple international stakeholders including consumers, clinicians and researchers. These results will directly inform the updated international PCOS guidelines. Limitations include the lack of RCTs precluding meta‐analysis, small participant numbers, as well as the high RoB of the included studies.

## 5 CONCLUSION

PCOS is a common, complex reproductive, metabolic and psychological condition which impacts many aspects of a woman's life. Here we have identified that only a few studies exist with all outcomes having low to very low certainty of evidence reflecting that research in PCOS has been underfunded and underprioritised, with limited high‐quality studies. Thus, the pending 2023 updated PCOS guidelines encompassed evidence from the general population when making recommendations. Based on this combined data, COCP use in women with PCOS is recommended to provide *contraception, cycle regulation and to treat clinical hyperandrogenism*. Further studies are needed in PCOS to understand the nuances of COCP efficacy, safety and adverse effects in this common and neglected condition to promote individualised therapeutic interventions.

## AUTHOR CONTRIBUTIONS

This work is part of the ongoing 2023 update of the evidence‐based international PCOS guidelines. Aya Mousa and Chau Thien Tay were part of the evidence team and Helena Teede led the update of the PCOS guidelines. Helena Teede, Terhi Piltonen, Alexia Pena, Selma Witchel, and Daniela Romualdi were senior experts within the guideline group working with medical treatments in PCOS. Maria Forslund, Johanna Melin, and Simon Alesi performed the search, screening, RoB assessments and GRADE assessments. Maria Forslund and Johanna Melin did the data extraction. Maria Forslund drafted the first version of the manuscript. All authors revised the manuscript and approved the final version.

## CONFLICT OF INTEREST STATEMENT

The authors declare no conflict of interest.

## ETHICS STATEMENT

The protocol for the systematic review was registered prospectively in Prospero, CRD42022345640.

## Supporting information

Supporting information.

## Data Availability

Data will be made available to the editors of the journal for review or query upon request (The data underlying this article are available in the article and in its online Supporting Information Material).
