# Supplementary material for: Combined oral contraceptive pill compared with no medical treatment in the management of polycystic ovary syndrome: A systematic review
Source: Clin Endocrinol (Oxf). 2023 Mar 27;99(1):79–91. doi: 10.1111/cen.14913 (PMC10952804; doi:10.1111/cen.14913)
Supplement: Supplementary file 1 — Supporting information. [file CEN-99-79-s001.docx]

**Supplementary Table 1.** Search string for three questions for the PCOS guideline evidence (Is the oral contraceptive pill alone or in combination effective for management of hormonal and clinical PCOS features in adolescents and adults with PCOS?

Is metformin alone or in combination, effective for management of hormonal and clinical PCOS features and weight in adolescents and adults with PCOS?Are anti-androgen pharmacological agents alone or in combination, effective for management of hormonal and clinical PCOS features and weight in adolescents and adults with PCOS?

The search was done for three medical treatments simultaneously to align with the previous guideline.

*OVID Medline, All EBM, PsychInfo, EMBASE*

*1 exp polycystic ovary syndrome/*

*2 polycystic ovar*.mp.*

*3 poly-cystic ovar*.mp.*

*4 PCO*.mp.*

*5 (stein-leventhal or leventhal).mp.*

*6 anovulation/*

*7 anovulat*.mp.*

*8 oligo-ovulat*.mp.*

*9 oligoovulat*.mp.*

*10 (ovar* adj5 (sclerocystic or polycystic or poly-cystic or degenerat**

*or hyperandrogen* or hyper-androgen*)).mp.*

*11 or/1-10*

*12 exp Contraceptives, Oral/*

*13 ((Oral and contracept*) or OCP or COCP).tw.*

*14 exp Metformin/*

*15 Metformin*.tw.*

*16 exp Androgen Antagonists/*

*17 *Spironolactone/*

*18 *Finasteride/*

*19 (anti?androgen* or anti androgen or androgen antagonist* or*

*spironolactone or cyproterone acetate or finasteride or flutamide).mp.*

*20 exp Anti-Obesity Agents/*

*21 *Obesity/th [Therapy]*

*22 ((anti?obesity or anti obesity or weight loss) and (agent* or*

*drug*)).mp.*

*23 (orlistat or sibutramine).mp.*

*24 *Inositol/*

*25 (inositol* or myo?inositol* or myo inositol* or meso?inositol* or*

*meso inositol* or i-inositol* or epi?inositol* or epi inositol* or*

*chiro?inositol* or chiro inositol* or l-chiro?inositol* or l-chiro inositol*).mp.*

*26 or/12-25*

*27 search$.tw. or meta-analysis.mp. or meta-analysis.pt. or review.pt.*

*or di.xs. or associated.tw.*

*28 clinical trial.mp. or clinical trial.pt. or random.mp. or tu.xs.*

*29 27 or 28*

*30 11 and 26 and 29*

*CINAHL*

*S26 S11 AND S25*

*S25 S12 OR S13*

*OR S14 OR S15*

*OR S16 OR S17*

*OR S18 OR S19*

*OR S20 OR S21*

*OR S22 OR S23*

*OR S24*

*S24 (inositol* or*

*myo?inositol* or*

*meso?inositol* or*

*i-inositol* or epi?*

*inositol* or chiro?*

*inositol or l-chiro?*

*inositol*)*

*S23 (MH*

*"Inositol+")*

*S22 (orlistat or sibutramine)*

*S21 ((anti?obesity*

*or weight loss or*

*weight-loss) and*

*(agent* or drug*))*

*S20 (MH*

*"Antiobesity*

*Agents+")*

*S19 (anti?*

*androgen* or anti*

*androgen or*

*androgen*

*antagonist* or*

*spironolactone or*

*cyproterone*

*acetate or*

*finasteride or*

*flutamide)*

*S18 (MH*

*"Finasteride")*

*S17 (MH*

*"Spironolactone+")*

*S16 (MH "Androgen*

*Antagonists+")*

*S15 metformin**

*S14 (MH*

*"Metformin")*

*S13 ((Oral and*

*contracept*) or*

*OCP or COCP)*

*S12 (MH*

*"Contraceptives,*

*Oral+")*

*S11 S1 OR S2 OR*

*S3 OR S4 OR S5*

*OR S6 OR S7 OR*

*S8 OR S9 OR*

*S10*

*S10 ovar* N5*

*sclerocystic or*

*ovar* N5 polycystic*

*or ovar* N5 polycystic*

*or ovar* N5*

*degenerat* or*

*ovar* N5*

*hyperandrogen* or*

*ovar* N5 hyperandrogen**

*S9 oligoovulat**

*S8 oligo-ovulat**

*S7 SU anovulation*

*S6 SU ovarian*

*Cysts*

*S5 stein-leventhal*

*or Leventhal*

*S4 PCO**

*S3 poly-cystic*

*ovar**

*S2 polycystic*

*ovar**

*S1 SU polycystic*

*ovary syndrome*

| **Supplementary table 2 . Excluded studies (on full-text assessment)** | |
| --- | --- |
| **Reference** | **Reason** |
| Unknown. Effect of green tea pills and metformin versus placebo on the Nrf2-antioxidant system and proinflammatory cytokines, including IL-6 and TNF-a, in peripheral blood mononuclear cells of women with polycystic ovary syndrome: a single blind randomized clinica 2017. | **Wrong publication type.** |
| Effect of supplementation in treatment of women with polycystic ovary syndrome. Clinical trial of the effect of inofolic supplementation compared with metformin on parameters of mental health and oxidative stress in women with polycystic ovary syndrome 2017. | **Wrong publication type.** |
| Effect of inofolic supplementation in treatment of women with polycystic ovary syndrome. Clinical trial of the effect of inofolic supplementation compared with metformin on metabolic profiles and gene expression related to insulin and lipid in women with polycystic ovary syndrome 2017. | **Full text not obtainable.** |
| Comparison of oral contraceptives including Contrasmine, Etisterone and Desoceptive with Ovustop-L (LD) on clinical, biochemical and metabolic findings, and quality of life in women with polycystic ovary syndrome. A Randomized cross-over clinical trial to assess the effectiveness of oral contraceptives including Contrasmine, Etisterone and Desoceptive with Ovustop-L (LD) on clinical, biochemical and metabolic findings, and quality of life in women with polycystic o 2017. | **Full text not obtainable.** |
| The efficacy of Fennel infusion and cupping on ovarian failure. Comparison of ovarian cupping and fennel infusion with Metformin on oligomenorrhea and ovulation in women with polycystic ovarian syndrome: a clinical trial 2017. | **Full text not obtainable.** |
| Scientific Impact Paper No. 13: Metformin Therapy for the Management of Infertility in Women with Polycystic Ovary Syndrome. Obstetrician & Gynaecologist 2017, 19, 339-339, doi:10.1111/tog.12436. | **Wrong study design.** |
| Effect of using metformin on the incidence of gestational diabetes and preeclampsia in pregnant women with polycystic ovary. Effect of using metformin versus not using on the incidence of gestational diabetes and preeclampsia in pregnant women with polycystic ovary: A randomized clinical trial 2018. | **Wrong publication type** |
| ?Effects of myo-inositol on induction of ovulation. Comparison the effects of myo-inositol plus clomiphene citrate with metformin plus clomiphene citrate on induction of ovulation among patients with polycystic ovarian syndrome. 2018. | **Wrong publication type** |
| New strategies to lose weight for women with polycystic ovary syndrome. Novel strategies in weight loss in women with polycystic ovary syndrome: does the gut microbiome play a role? 2018. | **Wrong publication type** |
| A study to compare the efficacy of two drugs on the success of assisted reproductive therapy in women with polycystic ovarian syndrome and undergoing treatment with IVF. Randomised Control Trial comparing the effects of Metformin to Myoinositol on ART outcome in women with PCOS undergoing IVF cycles 2018. | **Wrong publication type** |
| A study to compare the efficacy and adverse effects of metformin versus myoinositol plus d-chiroinositol combination therapy in polycystic ovarian syndrome. A prospective randomised comparative study of metformin versus myoinositol plus d-chiroinositol combination therapy in polycystic ovarian syndrome 2019. | **Wrong publication type** |
| Effect of combined electroacupuncture and medical therapy on insulin resistance in polycystic ovary syndrome patients. Combination of electroacupuncture and pharmacological treatment in improving insulin resistance (HOMA-IR) in polycystic ovary syndrome patients: a double-blind randomized clinical trial 2020. | **Wrong study design** |
| Study to find Effects of Chandraprabha Vati(Ayurvedic Medicine) in Polycystic Ovarian Syndrome Characterised by Small cysts in ovary with irregular,Scanty menses and excess/unwanted hairs on Face,Thighs,Abdomen etc. &acirc;??Randomized controlled clinical trial to study the efficacy OF Chandraprabha vati in PCOS.&acirc;?? 2020. | **Wrong study design** |
| A clinical trial to study the effect of exercise and metformin on mitochondrial health in patients with polycystic ovarian syndrome (PCOS). To assess the efficacy of moderate-intensity exercise training and metformin on mitophagy and mitochondrial phenotype in patients with polycystic ovarian syndrome (PCOS) 2020. | **Wrong publication type** |
| ffect of oral contraceptives on levels of adipokines, and adiposity indices in women with polycystic ovary syndrome. A randomized clinical trial to compare the effectiveness of oral contraceptives containing levonorgestrel, desogestrel, cyproterone acetate, and drospirenone on levels of adipokines, and adiposity indices in women with polycystic ovary syndrome. 2020. | **Wrong publication type** |
| Effect of treatment by OCP on infertility in PCOD patients. The randomized, single -blinded clinical trial comparing OCP effect before frozen embryo transfer versus gonadotropin &acirc;&ldquo; releasing hormone agonist injection on improving the outcome of pregnancy in infertile patients with hyper androgenic poly 2020. | **Fulltext not obtainable** |
| Expression of concern: Comparison of myo-inositol and metformin on mental health parameters and biomarkers of oxidative stress in women with polycystic ovary syndrome: a randomized, double-blind, placebo-controlled trial The effects of fish oil omega-3 fatty acid supplementation on mental health parameters and metabolic status of patients with polycystic ovary syndrome: a randomized, double-blind, placebo-controlled trial. Journal of Psychosomatic Obstetrics & Gynecology 2020, 41, I-I, doi:10.1080/0167482X.2020.1842508 | **Wrong publication type** |
| Evaluation of therapeutic effects of crocina (saffron tablets) in patients with polycystic ovary syndrome: a randomized double-blind clinical trial. 2021. | **Wrong publication type** |
| A clinical study in women suffering from polycystic ovary syndrome (PCOS) to test the drug LPRI-424 (dienogest/ethinyl estradiol) during 9 months of treatment. A multicentre, phase III, double-blind, randomised clinical trial to assess the efficacy and safety of LPRI-424 (dienogest 2.00 mg / ethinyl estradiol 0.02 mg) in the treatment of polycystic ovary syndrome (PCOS) versus placebo during 9 cycles 2021. | **Wrong publication type** |
| A clinical trial to study the effect of myoinositol based therapy in combination with metformin as compared to metformin alone in women with polycystic ovarian syndrome. A Randomized Controlled Trial comparing Myoinositol based therapy in combination with Metformin versus Metformin monotherapy on the clinical, metabolic and hormonal parameters in Obese reproductive age women with Polycystic Ovarian Syndrome 2021. | **Wrong publication type** |
| Efficacy of very low carbohydrate diet combined with metformin in overweight / obese PCOS patients on changing of clinical phenotype, gut microbiota and plasma metabolome after treatment: a randomized, controlled clinical trial. 2021. | **Fulltext not obtainable** |
| A Phase II, randomised, multi-centric, multi-national clinical trial to evaluate the efficacy, tolerability, and safety of a fixed dose combination of Spironolactone, Pioglitazone & Metformin (SPIOMET) for adolescent girls and young adult women (AYAs) with polycystic ovary syndrome (PCOS). 2021. | **Wrong publication type** |
| Investigation on the efficacy and safety of Ceylon cinnamon (Cinammomum zeylanicum) compared to metformin in ameliorating symptoms of Polycystic Ovary Syndrome (PCOS): A randomized controlled trial. Investigation on the Efficacy and Safety of Ceylon Cinnamon (Cinammomum zeylanicum) and Metformin in Ameliorating Polycystic Ovary Syndrome (PCOS): A Randomized Controlled Trial 2022. | **Wrong comparator** |
| Abdalla, M.A.; Deshmukh, H.; Atkin, S.; Sathyapalan, T. The potential role of incretin-based therapies for polycystic ovary syndrome: a narrative review of the current evidence. Therapeutic Advances in Endocrinology and Metabolism 2021, 12, doi:<https://dx.doi.org/10.1177/2042018821989238>. | **Wrong study design** |
| Abdalmageed, O.S.; Farghaly, T.A.; Abdelaleem, A.A.; Abdelmagied, A.E.; Ali, M.K.; Abbas, A.M. Impact of Metformin on IVF Outcomes in Overweight and Obese Women With Polycystic Ovary Syndrome: A Randomized Double-Blind Controlled Trial. Reproductive sciences (Thousand Oaks, Calif.) 2019, 26, 1336-1342, doi:<https://dx.doi.org/10.1177/1933719118765985>. | **Wrong intervention** |
| Acmaz, G.; Cınar, L.; Acmaz, B.; Aksoy, H.; Kafadar, Y.T.; Madendag, Y.; Ozdemir, F.; Sahin, E.; Muderris, I. The Effects of Oral Isotretinoin in Women with Acne and Polycystic Ovary Syndrome. BioMed research international 2019, 10.1155/2019/2513067, 1-5, doi:10.1155/2019/2513067. | **Wrong study design** |
| Advani, K.; Batra, M.; Tajpuriya, S.; Gupta, R.; Saraswat, A.; Nagar, H.D.; Makwana, L.; Kshirsagar, S.; Kaul, P.; Ghosh, A.K., et al. Efficacy of combination therapy of inositols, antioxidants and vitamins in obese and non-obese women with polycystic ovary syndrome: an observational study. Journal of Obstetrics & Gynaecology 2020, 40, 96-101, doi:10.1080/01443615.2019.1604644. | **Wrong study design** |
| Ahc, M. What Are the Roles of the Combined Oral Contraceptive Pill and Metformin in the Management of Polycystic Ovary Syndrome? OB/GYN Clinical Alert 2020, 36, N.PAG-N.PAG. | **Wrong study design** |
| Ainehchi, N.; Khaki, A.; Ouladsahebmadarek, E.; Hammadeh, M.; Farzadi, L.; Farshbaf-Khalili, A.; Asnaashari, S.; Khamnei, H.J.; Khaki, A.A.; Shokoohi, M. The effect of clomiphene citrate, herbal mixture, and herbal mixture along with clomiphene citrate on clinical and para-clinical parameters in infertile women with polycystic ovary syndrome: A randomized controlled clinical trial. Archives of Medical Science 2020, 16, 1304-1318, doi:<https://dx.doi.org/10.5114/AOMS.2020.93271>. | **Wrong intervention** |
| Akhtar, T.; Shaikh, F.; Basma; Ahmed, W.U.N.; Lashari, S.; Bhatti, N. Comparison of myoinositol versus combination of metformin and myoinositol in ovulation induction in polycystic ovarian syndrome. Pakistan Journal of Medical and Health Sciences 2021, 15, 1494-1496, doi:<http://dx.doi.org/10.53350/pjmhs211561494>. | **Wrong outcome** |
| Alalami, H.; Sathyapalan, T.; Atkin, S.L. Cardiovascular profile of pharmacological agents used for the management of polycystic ovary syndrome. Therapeutic Advances in Endocrinology and Metabolism 2019, 10, doi:<http://dx.doi.org/10.1177/2042018818805674>. | **Wrong study design** |
| Alalfy, M.; Rashwan, A.S.S.A.; Hussein, M.; Bakry, A.; Eid, A.; Eid, M.M. The Use of N-Acetyl Cysteine Versus Chromium Picolinate as an Adjuvant to Clomiphene Citrate and Metformin in PCOS Women to Improve Ovulation Induction and Insulin Resistance: A Pilot Randomized Controlled Trial. Current Women's Health Reviews 2022, 18, e241221192204, doi:<https://dx.doi.org/10.2174/1573404817666210310164353>. | **Wrong comparator** |
| Alhussain, F.; Alruthia, Y.; Al-Mandeel, H.; Bellahwal, A.; Alharbi, F.; Almogbel, Y.; Awwad, O.; Dala'een, R.; Alharbi, F.A. Metformin improves the depression symptoms of women with polycystic ovary syndrome in a lifestyle modification program. Patient Preference and Adherence 2020, 14, 737-746, doi:<http://dx.doi.org/10.2147/PPA.S244273>. | **Wrong study design** |
| Ali, D.-E.S.; Shah, M.; Ali, A.; Malik, M.O.; Rehman, F.; Badshah, H.; Ehtesham, E.; Vitale, S.G. Treatment with Metformin and Combination of Metformin Plus Pioglitazone on Serum Levels of IL-6 and IL-8 in Polycystic Ovary Syndrome: A Randomized Clinical Trial. Hormone and metabolic research = Hormon- und Stoffwechselforschung = Hormones et metabolisme 2019, 51, 714-722, doi:<https://dx.doi.org/10.1055/a-1018-9606>. | **Fulltext not obtainable** |
| Almalki, H.H.; Alshibani, T.M.; Alhifany, A.A.; Almohammed, O.A. Comparative efficacy of statins, metformin, spironolactone and combined oral contraceptives in reducing testosterone levels in women with polycystic ovary syndrome: a network meta-analysis of randomized clinical trials. BMC women's health 2020, 20, 1-6, doi:10.1186/s12905-020-00919-5. | **Wrong intervention** |
| Amiri, M.; Kabir, A.; Nahidi, F.; Shekofteh, M.; Ramezani Tehrani, F. Effects of combined oral contraceptives on the clinical and biochemical parameters of hyperandrogenism in patients with polycystic ovary syndrome: a systematic review and meta-analysis. European journal of contraception & reproductive health care 2018, 23, 64-77, doi:10.1080/13625187.2018.1435779. | **Wrong comparator** |
| Amiri, M.; Nahidi, F.; Yarandi, R.B.; Khalili, D.; Tohidi, M.; Tehrani, F.R. Effects of oral contraceptives on the quality of life of women with polycystic ovary syndrome: a crossover randomized controlled trial. Health & Quality of Life Outcomes 2020, 18, N.PAG-N.PAG, doi:10.1186/s12955-020-01544-4. | **Wrong population** |
| Amiri, M.; Ramezani Tehrani, F.; Nahidi, F.; Kabir, A.; Azizi, F.; Carmina, E. Effects of oral contraceptives on metabolic profile in women with polycystic ovary syndrome: A meta-analysis comparing products containing cyproterone acetate with third generation progestins. Metabolism: clinical and experimental 2017, 73, 22-35, doi:<https://dx.doi.org/10.1016/j.metabol.2017.05.001>. | **Wrong comparator** |
| Amiri, M.; Tehrani, F.R.; Nahidi, F.; Kabir, A.; Azizi, F. Comparing the Effects of Combined Oral Contraceptives Containing Progestins With Low Androgenic and Antiandrogenic Activities on the Hypothalamic-Pituitary-Gonadal Axis in Patients With Polycystic Ovary Syndrome: Systematic Review and Meta-Analysis. Journal of Medical Internet Research 2018, 20, 1-1, doi:10.2196/resprot.9024. | **Wrong comparator** |
| Amirkhanloo, F.; Esmaeilzadeh, S.; Mirabi, P.; Abedini, A.; Amiri, M.; Saghebi, R.; Golsorkhtabaramiri, M. Comparison of Foeniculum Vulgare versus metformin on insulin resistance and anthropometric indices of women with polycystic ovary, an open-label controlled trial study. Obesity Medicine 2022, 31, 100401, doi:<https://dx.doi.org/10.1016/j.obmed.2022.100401>. | **Wrong comparator** |
| Ammar, I.M.M.; Salem, M.A.A. Amelioration of polycystic ovary syndrome-related disorders by supplementation of thymoquinone and metformin. Middle East Fertility Society Journal 2021, 26, 29, doi:<http://dx.doi.org/10.1186/s43043-021-00076-1>. | **Wrong comparator** |
| Andræ, F.; Abbott, D.; Stridsklev, S.; Schmedes, A.V.; Odsæter, I.H.; Vanky, E.; Salvesen, Ø. Sustained Maternal Hyperandrogenism During PCOS Pregnancy Reduced by Metformin in Non-obese Women Carrying a Male Fetus. Journal of Clinical Endocrinology & Metabolism 2020, 105, 1-9, doi:10.1210/clinem/dgaa605. | **Wrong population** |
| Anonymous. Metformin Therapy for the Management of Infertility in Women with Polycystic Ovary Syndrome: Scientific Impact Paper No. 13. BJOG : an international journal of obstetrics and gynaecology 2017, 124, e306-e313, doi:<https://dx.doi.org/10.1111/1471-0528.14764>. | **Wrong study design** |
| Anonymous. Screening and Management of the Hyperandrogenic Adolescent: ACOG Committee Opinion, Number 789. Obstetrics and gynecology 2019, 134, e106-e114, doi:<https://dx.doi.org/10.1097/AOG.0000000000003475>. | **Wrong study design** |
| Armanini, D.; Boscaro, M.; Bordin, L.; Sabbadin, C. Controversies in the Pathogenesis, Diagnosis and Treatment of PCOS: Focus on Insulin Resistance, Inflammation, and Hyperandrogenism. International journal of molecular sciences 2022, 23, doi:<https://dx.doi.org/10.3390/ijms23084110>. | **Wrong study design** |
| Artini, P.G.; Obino, M.E.R.; Sergiampietri, C.; Pinelli, S.; Papini, F.; Casarosa, E.; Cela, V. PCOS and pregnancy: a review of available therapies to improve the outcome of pregnancy in women with polycystic ovary syndrome. Expert review of endocrinology & metabolism 2018, 13, 87-98, doi:<https://dx.doi.org/10.1080/17446651.2018.1431122> | **Wrong study design** |
| Arya, S.; Hansen, K.R.; Wild, R.A. Metformin, rosiglitazone, or both for obese women with polycystic ovary syndrome? Fertility & Sterility 2020, 113, 87-88, doi:10.1016/j.fertnstert.2019.10.006. | **Wrong study design** |
| Asanidze, E.; Kristesashvili, J.; Pkhaladze, L.; Khomasuridze, A. The value of anti-Mullerian hormone in the management of polycystic ovary syndrome in adolescents. Gynecological endocrinology : the official journal of the International Society of Gynecological Endocrinology 2019, 35, 974-977, doi:<https://dx.doi.org/10.1080/09513590.2019.1616689>. | **Wrong comparator** |
| Ashok Kumar, M.; Samuel Gideon George, P.; Dasari, A.; Shanmugasundaram, P. A single-blinded randomized trial to evaluate the efficacy of N-acetyl cysteine over metformin in patients with polycystic ovarian syndrome. Drug Invention Today 2018, 10, 241-243. | **Wrong comparator** |
| Aversa, A.; La Vignera, S.; Rago, R.; Gambineri, A.; Nappi, R.E.; Calogero, A.E.; Ferlin, A. Fundamental concepts and novel aspects of polycystic ovarian syndrome: Expert consensus resolutions. Frontiers in endocrinology 2020, 11, 516, doi:<https://dx.doi.org/10.3389/fendo.2020.00516>. | **Wrong study design** |
| Azizi Kutenaei, M.; Hosseini Teshnizi, S.; Ghaemmaghami, P.; Eini, F.; Roozbeh, N. The effects of myo-inositol vs. metformin on the ovarian function in the polycystic ovary syndrome: a systematic review and meta-analysis. European review for medical and pharmacological sciences 2021, 25, 3105-3115, doi:<https://dx.doi.org/10.26355/eurrev_202104_25565>. | **Wrong comparator** |
| Bahadur, A.; Yadav, A.; Chaturvedi, J.; Mundhra, R.; Rajput, R.; Naithani, M.; Bhattacharya, N.; Prerna, J.; Kumari, S.; Verma, N., et al. Effect of two different doses of Vitamin D supplementation on clinical, metabolic and hormonal profiles of Insulin-resistant PCOS patients: a Randomized Controlled Trial. Human reproduction (Oxford, England) 2020, 35, i451. | **Wrong comparator** |
| Bahman, M.; Hajimehdipoor, H.; Bioos, S.; Hashem-Dabaghian, F.; Afrakhteh, M.; Tansaz, M. Effect of aslagh capsule, a traditional compound herbal product on oligomenorrhea in patients with polycystic ovary syndrome: A three-arm, open-label, randomized, controlled trial. Galen Medical Journal 2019, 8, 1261, doi:<http://dx.doi.org/10.31661/gmj.v0i0.1261>. | **Wrong comparator** |
| Baldani, D.P.; Skrgatic, L.; Ougouag, R.; Kasum, M. The cardiometabolic effect of current management of polycystic ovary syndrome: strategies of prevention and treatment. Gynecological endocrinology : the official journal of the International Society of Gynecological Endocrinology 2018, 34, 87-91, doi:<https://dx.doi.org/10.1080/09513590.2017.1381681>. | **Wrong study design** |
| Bansal, Y.; Sharma, N. Effect of metformin on levels of androgen in obese women having PCOS with & without Mg supplementation: a randomized control trial. Clinica chimica acta 2022, 530, S412, doi:<https://doi.org/10.1016/j.cca.2022.04.441>. | **Wrong publication type** |
| Battaglia, C.; Battaglia, B.; Casadio, P.; Rizzo, R.; Artini, P.G. Metformin metabolic and vascular effects in normal weight hyperinsulinemic polycystic ovary syndrome patients treated with contraceptive vaginal ring. A pilot study. Gynecological endocrinology : the official journal of the International Society of Gynecological Endocrinology 2020, 36, 1062-1069, doi:<https://dx.doi.org/10.1080/09513590.2020.1770213>. | **Wrong comparator** |
| Behboudi-Gandevani, S.; Abtahi, H.; Saadat, N.; Tohidi, M.; Ramezani Tehrani, F. Effect of phlebotomy versus oral contraceptives containing cyproterone acetate on the clinical and biochemical parameters in women with polycystic ovary syndrome: a randomized controlled trial. Journal of ovarian research 2019, 12, 78, doi:<https://dx.doi.org/10.1186/s13048-019-0554-9>. | **Wrong comparator** |
| Bhide, P.; Pundir, J.; Gudi, A.; Shah, A.; Homburg, R.; Acharya, G. The effect of myo-inositol/di-chiro-inositol on markers of ovarian reserve in women with PCOS undergoing IVF/ICSI: A systematic review and meta-analysis. Acta obstetricia et gynecologica Scandinavica 2019, 98, 1235-1244, doi:10.1111/aogs.13625. | **Wrong intervention** |
| Bidhendi Yarandi, R.; Behboudi-Gandevani, S.; Amiri, M.; Ramezani Tehrani, F. Metformin therapy before conception versus throughout the pregnancy and risk of gestational diabetes mellitus in women with polycystic ovary syndrome: A systemic review, meta-analysis and meta-regression. Diabetology and Metabolic Syndrome 2019, 11, 58, doi:<http://dx.doi.org/10.1186/s13098-019-0453-7>. | **Wrong outcome** |
| Bjekic-Macut, J.; Vukasin, T.; Velija-Asimi, Z.; Burekovic, A.; Zdravkovic, M.; Andric, Z.; Brankovic, M.; Crevar-Marinovic, S.; Madic, T.; Stanojlovic, O., et al. Polycystic Ovary Syndrome: A Contemporary Clinical Approach. Current pharmaceutical design 2021, 27, 3812-3820, doi:<https://dx.doi.org/10.2174/1381612827666210119104721>. | **Wrong study design** |
| Bordewijk, E.M.; Nahuis, M.; Costello, M.F.; Van der Veen, F.; Tso, L.O.; Mol, B.W.J.; van Wely, M. Metformin during ovulation induction with gonadotrophins followed by timed intercourse or intrauterine insemination for subfertility associated with polycystic ovary syndrome. The Cochrane database of systematic reviews 2017, 1, CD009090, doi:<https://dx.doi.org/10.1002/14651858.CD009090.pub2>. | **Wrong intervention** |
| Boyd, M.; Ziegler, J. Polycystic Ovary Syndrome, Fertility, Diet, and Lifestyle Modifications: A Review of the Current Evidence. Topics in Clinical Nutrition 2019, 34, 14-30, doi:10.1097/TIN.0000000000000161. | **Wrong study design** |
| Burgart, J.M. Polycystic Ovary Disease and Obesity: Leptin, Weight-loss Medication, and Bariatric Surgery. Clinical Obstetrics & Gynecology 2021, 64, 90-95, doi:10.1097/GRF.0000000000000599. | **Wrong study design** |
| Cai, M.; Zhang, Y.; Qu, S.; Zhang, M. The safety and efficacy of canagliflozin in women with polycystic ovary syndrome: a randomized control trial. Diabetes 2021, 70, doi:<https://doi.org/10.2337/db21-132-LB>. | **Wrong publication type** |
| Campbell, A. What to Know About Metformin. Diabetes Self-Management 2019, 36, 20-21. | **Wrong study design** |
| Cantelmi, T.; Lambiase, E.; Unfer, V.R.; Gambioli, R.; Unfer, V. Inositol treatment for psychological symptoms in Polycystic Ovary Syndrome women. European review for medical and pharmacological sciences 2021, 25, 2383-2389, doi:<https://dx.doi.org/10.26355/eurrev_202103_25278>. | **Wrong study design** |
| Cao, Q.; Hu, Y.; Fu, J.; Huang, X.; Wu, L.; Zhang, J.; Huang, W. Gestational metformin administration in women with polycystic ovary syndrome: A systematic review and meta‐analysis of randomized control studies. Journal of Obstetrics & Gynaecology Research 2021, 47, 4148-4157, doi:10.1111/jog.15044. | **Wrong outcome** |
| Cao, Y.; Chen, H.; Zhao, D.; Zhang, L.; Yu, X.; Zhou, X.; Liu, Z. The efficacy of Tung's acupuncture for sex hormones in polycystic ovary syndrome: A randomized controlled trial. Complementary therapies in medicine 2019, 44, 182-188, doi:10.1016/j.ctim.2019.04.016. | **Wrong intervention** |
| Cao, Y.; Zhang, L.; Zhao, D.; Liu, Z. [DONG's extraordinary acupoints for the ovarian function of polycystic ovary syndrome:a randomized controlled pilot trial]. Zhongguo zhen jiu = Chinese acupuncture & moxibustion 2017, 37, 710-714, doi:<https://dx.doi.org/10.13703/j.0255-2930.2017.07.007>. | **Wrong language** |
| Capozzi, A.; Scambia, G.; Lello, S. Polycystic ovary syndrome (PCOS) and adolescence: How can we manage it? European Journal of Obstetrics & Gynecology & Reproductive Biology 2020, 250, 235-240, doi:10.1016/j.ejogrb.2020.04.024 | **Wrong study design** |
| Cappelli, V.; Musacchio, M.C.; Bulfoni, A.; Morgante, G.; De Leo, V. Natural molecules for the therapy of hyperandrogenism and metabolic disorders in PCOS. European review for medical and pharmacological sciences 2017, 21, 15-29. | **Wrong study design** |
| Carmina, E.; Azziz, R.; Bergfeld, W.; Escobar-Morreale, H.F.; Futterweit, W.; Huddleston, H.; Lobo, R.; Olsen, E. Female Pattern Hair Loss and Androgen Excess: A Report From the Multidisciplinary Androgen Excess and PCOS Committee. The Journal of clinical endocrinology and metabolism 2019, 104, 2875-2891, doi:<https://dx.doi.org/10.1210/jc.2018-02548>. | **Wrong study design** |
| Carmina, E.; Dreno, B.; Lucky, W.A.; Agak, W.G.; Dokras, A.; Kim, J.J.; Lobo, R.A.; Tehrani, F.R.; Dumesic, D. Female Adult Acne and Androgen Excess: A Report From the Multidisciplinary Androgen Excess and PCOS Committee. Journal of the Endocrine Society 2022, 6, 1-11, doi:10.1210/jendso/bvac003. | **Wrong intervention** |
| Carvalho, L.M.L.; Ferreira, C.N.; Candido, A.L.; Reis, F.M.; Soter, M.O.; Sales, M.F.; Silva, I.F.O.; Nunes, F.F.C.; Gomes, K.B. Metformin reduces total microparticles and microparticles-expressing tissue factor in women with polycystic ovary syndrome. Archives of gynecology and obstetrics 2017, 296, 617-621, doi:<https://dx.doi.org/10.1007/s00404-017-4471-0>. | **Wrong study design** |
| Casey, G. Metformin - for more than just diabetes? Kai Tiaki Nursing New Zealand 2019, 25, 20-20. | **Wrong study design** |
| Chatzis, P.; Tziomalos, K.; Pratilas, G.C.; Makris, V.; Sotiriadis, A.; Dinas, K. The Role of Antiobesity Agents in the Management of Polycystic Ovary Syndrome. Folia medica 2018, 60, 512-520, doi:<https://dx.doi.org/10.2478/folmed-2018-0036>. | **Wrong study design** |
| Chen, M.; Yang, P.; Chen, H.; Chen, S.; Ho, H. The efficacy of long-term metformin treatment in women with polycystic ovary syndrome. Fertility & Sterility 2017, 108, e245-e246, doi:10.1016/j.fertnstert.2017.07.738. | **Wrong study design** |
| Chen, X.; He, S.; Wang, D. Effects of metformin on body weight in polycystic ovary syndrome patients: model-based meta-analysis. Expert review of clinical pharmacology 2021, 14, 121-130, doi:<https://dx.doi.org/10.1080/17512433.2021.1863788>. | **Wrong study design** |
| hen, Y.; Li, M.; Deng, H.; Wang, S.; Chen, L.; Li, N.; Xu, D.; Wang, Q. Impact of metformin on C-reactive protein levels in women with polycystic ovary syndrome: A meta-analysis. Oncotarget 2017, 8, 35425-35434, doi:<http://dx.doi.org/10.18632/oncotarget.16019>. | **Wrong comparator** |
| Chen, Z.; Tan, J.; Wang, H.; Zheng, B.; Liu, J.; Hao, G.; Guo, Z.; Sun, Z.; Yu, Q. A Randomized Cohort Study: is It Worth the Time to Receive Antiandrogenic Pretreatment Before Ovulation Induction for Women With Polycystic Ovary Syndrome? Frontiers in endocrinology 2022, 13, doi:<https://doi.org/10.3389/fendo.2022.813188>. | **Wrong comparator** |
| Cignarella, A.; Mioni, R.; Sabbadin, C.; Dassie, F.; Parolin, M.; Vettor, R.; Barbot, M.; Scaroni, C. Pharmacological Approaches to Controlling Cardiometabolic Risk in Women with PCOS. International journal of molecular sciences 2020, 21, doi:<https://dx.doi.org/10.3390/ijms21249554>. | **Wrong study design** |
| Condorelli, R.A.; Calogero, A.E.; Di Mauro, M.; Mongioi, L.M.; Cannarella, R.; Rosta, G.; La Vignera, S. Androgen excess and metabolic disorders in women with PCOS: beyond the body mass index. Journal of endocrinological investigation 2018, 41, 383-388, doi:<https://dx.doi.org/10.1007/s40618-017-0762-3>. | **Wrong study design** |
| Costello, M.F.; Misso, M.L.; Balen, A.; Boyle, J.; Devoto, L.; Garad, R.M.; Hart, R.; Johnson, L.; Jordan, C.; Legro, R.S., et al. Evidence summaries and recommendations from the international evidence-based guideline for the assessment and management of polycystic ovary syndrome: Assessment and treatment of infertility. Human Reproduction Open 2019, 2019, hoy021, doi:<http://dx.doi.org/10.1093/hropen/hoy021>. | **Wrong outcome** |
| Costello, M.F.; Misso, M.L.; Balen, A.; Boyle, J.; Devoto, L.; Garad, R.M.; Hart, R.; Johnson, L.; Jordan, C.; Legro, R.S., et al. A brief update on the evidence supporting the treatment of infertility in polycystic ovary syndrome. Australian & New Zealand journal of obstetrics & gynaecology 2019, 59, 867-873, doi:10.1111/ajo.13051. | **Wrong study design** |
| Craig, M.; Temples, H.S.; Weir, B. Polycystic Ovarian Syndrome in Adolescents: Early Diagnosis and Intervention. Journal of Pediatric Healthcare 2020, 34, 166-170, doi:10.1016/j.pedhc.2019.11.007. | **Wrong study design** |
| rellin, H. What are the most effective oral medications for ovulation induction in women with PCOS? Evidence-Based Practice 2022, 25, 26-27, doi:10.1097/EBP.0000000000001408. | **Wrong study design** |
| Crouch, R.; Hamilton, J.; Raymond, T. Is metformin effective for treating infertility associated with PCOS? Evidence-Based Practice 2022, 25, 35-36, doi:10.1097/EBP.0000000000001328. | **Wrong study design** |
| Cui, N.; Feng, X.; Zhao, Z.; Zhang, J.; Xu, Y.; Wang, L.; Hao, G. Restored Plasma Anandamide and Endometrial Expression of Fatty Acid Amide Hydrolase in Women With Polycystic Ovary Syndrome by the Combination Use of Diane-35 and Metformin. Clinical therapeutics 2017, 39, 751-758, doi:10.1016/j.clinthera.2017.02.007. | **Wrong study design** |
| Daneshjou, D.; Mehranjani, M.S.; Zadehmodarres, S.; Shariatzadeh, S.M.A.; Mofarahe, Z.S. Sitagliptin/metformin improves the fertilization rate and embryo quality in polycystic ovary syndrome patients through increasing the expression of GDF9 and BMP15: a new alternative to metformin (a randomized trial). Journal of reproductive immunology 2022, 150, doi:<https://doi.org/10.1016/j.jri.2022.103499>. | **Wrong intervention** |
| Daneshjou, D.; Soleimani Mehranjani, M.; Zadeh Modarres, S.; Shariatzadeh, M.A. Sitagliptin/Metformin: A New Medical Treatment in Polycystic Ovary Syndrome. Trends in endocrinology and metabolism: TEM 2020, 31, 890-892, doi:<https://dx.doi.org/10.1016/j.tem.2020.09.002>. | **Wrong study design** |
| Daneshjou, D.; Zadeh Modarres, S.; Soleimani Mehranjani, M.; Shariat Zadeh, S.M.A. Comparing the effect of sitagliptin and metformin on the oocyte and embryo quality in classic PCOS patients undergoing ICSI. Irish journal of medical science 2021, 190, 685-692, doi:<https://dx.doi.org/10.1007/s11845-020-02320-5>. | **Wrong intervention** |
| de Medeiros, S.F. Risks, benefits size and clinical implications of combined oral contraceptive use in women with polycystic ovary syndrome. Reproductive biology and endocrinology : RB&E 2017, 15, 93, doi:<https://dx.doi.org/10.1186/s12958-017-0313-y>. | **Wrong study design** |
| de Medeiros, S.F.; Medeiros, M.A.S.d.; Santos, N.d.S.; Barbosa, B.B.; Yamamoto, M.M.W. Combined Oral Contraceptive Effects on Low-Grade Chronic Inflammatory Mediators in Women with Polycystic Ovary Syndrome: A Systematic Review and Meta-Analysis. International Journal of Inflammation 2018, 10.1155/2018/9591509, 1-13, doi:10.1155/2018/9591509. | **Wrong comparator** |
| Della Corte, L.; La Rosa, V.L.; Rapisarda, A.M.C.; Valenti, G.; Morra, I.; Boccellino, A.; Zizolfi, B.; Santangelo, F.; de Rosa, N.; Sapia, F., et al. Current evidences and future perspectives on patient-oriented treatments for polycystic ovary syndrome: An overview. Italian Journal of Gynaecology and Obstetrics 2018, 30, 7-20, doi:<http://dx.doi.org/10.14660/2385-0868-81>. | **Wrong study design** |
| Deng, Y.; Wang, Y.-F.; Zhu, S.-Y.; Ma, X.; Xue, W.; Ma, R.-L.; Sun, A.-J. Is There An Advantage of Using Dingkun Pill () alone or in Combination with Diane-35 for Management of Polycystic Ovary Syndrome? A Randomized Controlled Trial. Chinese journal of integrative medicine 2020, 26, 883-889, doi:<https://dx.doi.org/10.1007/s11655-020-3097-4>. | **Wrong comparator** |
| Devi, N.; Boya, C.; Chhabra, M.; Bansal, D. N-acetyl-cysteine as adjuvant therapy in female infertility: a systematic review and meta-analysis. Journal of basic and clinical physiology and pharmacology 2020, 32, 899-910, doi:<https://dx.doi.org/10.1515/jbcpp-2020-0107>. | **Wrong population** |
| Devi, N.; Boya, C.; Chhabra, M.; Bansal, D. N-acetyl-cysteine as adjuvant therapy in female infertility: a systematic review and meta-analysis. Journal of Basic & Clinical Physiology & Pharmacology 2021, 32, 899-910, doi:10.1515/jbcpp-2020-0107. | **Wrong intervention** |
| Diaz, M.; Bassols, J.; Lopez-Bermejo, A.; De Zegher, F.; Ibanez, L. Circulating miR-451a: a biomarker to guide diagnosis and treatment of polycystic ovary syndrome in adolescent girls. Hormone research in paediatrics 2019, 91, 117, doi:<https://doi.org/10.1159/000501868>. | **Wrong outcome** |
| Díaz, M.; Bassols, J.; López-Bermejo, A.; de Zegher, F.; Ibáñez, L. Low Circulating Levels of miR-451a in Girls with Polycystic Ovary Syndrome: Different Effects of Randomized Treatments. Journal of Clinical Endocrinology & Metabolism 2019, 10.1210/clinem/dgz204, N.PAG-N.PAG, doi:10.1210/clinem/dgz204. | **Wrong outcome** |
| Dm, S.M. Abstract #1184447: effects of vitamin D supplementation on metabolic and endocrine abnormalities in polycystic ovary syndrome. Endocrine practice 2022, 28, S124, doi:<https://doi.org/10.1016/j.eprac.2022.03.292>. | **Wrong publication type** |
| Dodd, J.M.; Grivell, R.M.; Deussen, A.R.; Hague, W.M. Metformin for women who are overweight or obese during pregnancy for improving maternal and infant outcomes. The Cochrane database of systematic reviews 2018, 7, CD010564, doi:<https://dx.doi.org/10.1002/14651858.CD010564.pub2>. | **Wrong population** |
| Doi, S.A.R.; Furuya-Kanamori, L.; Toft, E.; Musa, O.A.H.; Islam, N.; Clark, J.; Thalib, L. Metformin in pregnancy to avert gestational diabetes in women at high risk: Meta-analysis of randomized controlled trials. Obesity reviews : an official journal of the International Association for the Study of Obesity 2020, 21, e12964, doi:<https://dx.doi.org/10.1111/obr.12964>. | **Wrong outcome** |
| Dubois, W.I.L. METFORMIN: THE UNAUTHORIZED BIOGRAPHY. Diabetes Self-Management 2022, 39, 62-67. | **Wrong publication type** |
| Duguech, L.M.M.; Legro, R.S. Pharmacologic Treatment of Polycystic Ovary Syndrome: Alternate and Future Paths. Seminars in reproductive medicine 2017, 35, 326-343, doi:<https://dx.doi.org/10.1055/s-0037-1603729>. | **Wrong study design** |
| Dwivedi, A.N.D.; Ganesh, V.; Shukla, R.C.; Jain, M.; Kumar, I. Colour Doppler evaluation of uterine and ovarian blood flow in patients of polycystic ovarian disease and post-treatment changes. Clinical radiology 2020, 75, 772-779, doi:10.1016/j.crad.2020.05.023. | **Wrong outcome** |
| El Sharkwy, I.; Sharaf El-Din, M. l-Carnitine plus metformin in clomiphene-resistant obese PCOS women, reproductive and metabolic effects: a randomized clinical trial. Gynecological Endocrinology 2019. | **Wrong intervention** |
| Elkind-Hirsch, K.E.; Paterson, M.; Seidemann, E.; Gutowski, H. Body mass index does not affect suppression of hyperandrogenism but does impact carbohydrate metabolism during low-dose folate-supplemented ethinyl estradiol/drospirenone oral contraceptive therapy in women with polycystic ovary syndrome. Journal of Reproductive Medicine 2017, 62, 357. | **Wrong study design** |
| Elvir Zelaya, R.; Carbia, C.D.O.; Chong, A.B.D.O.; Hahn, K.D.O. In women with polycystic ovary syndrome, does pioglitazone decrease testosterone more than metformin? Evidence-Based Practice 2021, 24, 35-38, doi:10.1097/EBP.0000000000000892. | **Wrong study design** |
| Facchinetti, F.; Appetecchia, M.; Aragona, C.; Bevilacqua, A.; Bezerra Espinola, M.S.; Bizzarri, M.; D'Anna, R.; Dewailly, D.; Diamanti-Kandarakis, E.; Hernandez Marin, I., et al. Experts' opinion on inositols in treating polycystic ovary syndrome and non-insulin dependent diabetes mellitus: a further help for human reproduction and beyond. Expert opinion on drug metabolism & toxicology 2020, 16, 255-274, doi:<https://dx.doi.org/10.1080/17425255.2020.1737675>. | **Wrong study design** |
| Facchinetti, F.; Orru, B.; Grandi, G.; Unfer, V. Short-term effects of metformin and myo-inositol in women with polycystic ovarian syndrome (PCOS): a meta-analysis of randomized clinical trials. Gynecological endocrinology : the official journal of the International Society of Gynecological Endocrinology 2019, 35, 198-206, doi:<https://dx.doi.org/10.1080/09513590.2018.1540578>. | **Wrong comparator** |
| Fang, F.; Ni, K.; Cai, Y.; Shang, J.; Zhang, X.; Xiong, C. Effect of vitamin D supplementation on polycystic ovary syndrome: A systematic review and meta-analysis of randomized controlled trials. Complementary therapies in clinical practice 2017, 26, 53-60, doi:10.1016/j.ctcp.2016.11.008. | **Wrong intervention** |
| Farhadian, M.; Barati, S.; Mahmoodi, M.; Barati Mosleh, A.; Yavangui, M. Comparison of green tea and metformin effects on anthropometric indicators in women with polycystic ovarian syndrome: A clinical trial study. Journal of Reports in Pharmaceutical Sciences 2020, 9, 97, doi:<https://doi.org/10.4103/jrptps.JRPTPS1419>. | **Wrong comparator** |
| Fatima, A.; Khan, S.A.; Saifuddin, Z.; Aslam, R. Comparison of efficacy of clomiphene citrate alone and with metformin for treatment of infertility in polycystic ovarian syndrome. Rawal Medical Journal 2018, 43, 285-288. | **Wrong intervention** |
| Ferrer, M.J.; Silva, A.F.; Abruzzese, G.A.; Velazquez, M.E.; Motta, A.B. Lipid Metabolism and Relevant Disorders to Female Reproductive Health. Current medicinal chemistry 2021, 28, 5625-5647, doi:<https://dx.doi.org/10.2174/0929867328666210106142912> | **Wrong study design** |
| Figurova, J.; Dravecka, I.; Petrikova, J.; Javorsky, M.; Lazurova, I. The effect of alfacalcidiol and metformin on metabolic disturbances in women with polycystic ovary syndrome. Hormone molecular biology and clinical investigation 2017, 29, 85-91, doi:https://dx.doi.org/10.1515/hmbci-2016-0039. | **Wrong comparator** |
| Fougner, S.L.; Vanky, E.; Lovvik, T.S.; Carlsen, S.M. No impact of gestational diabetes mellitus on pregnancy complications in women with PCOS, regardless of GDM criteria used. PloS one 2021, 16, e0254895, doi:<https://dx.doi.org/10.1371/journal.pone.0254895>. | **Wrong outcome** |
| Fruzzetti, F.; Perini, D.; Russo, M.; Bucci, F.; Gadducci, A. Comparison of two insulin sensitizers, metformin and myo-inositol, in women with polycystic ovary syndrome (PCOS). Gynecological Endocrinology 2017, 33, 39, doi:<https://doi.org/10.1080/09513590.2016.1236078>. | **Wrong comparator** |
| Fujita, Y.; Inagaki, N. Metformin: clinical topics and new mechanisms of action. Diabetology International 2017, 8, 4-6, doi:<http://dx.doi.org/10.1007/s13340-016-0300-0>. | **Wrong study design** |
| Gadalla, M.A.; Norman, R.J.; Tay, C.T.; Hiam, D.S.; Melder, A.; Pundir, J.; Thangaratinam, S.; Teede, H.J.; Mol, B.W.J.; Moran, L.J. Medical and Surgical Treatment of Reproductive Outcomes in Polycystic Ovary Syndrome: An Overview of Systematic Reviews. International Journal of Fertility & Sterility 2020, 13, 257-270, doi:10.22074/ijfs.2020.5608. | **Wrong study design** |
| Garad, R.M.; Teede, H.J. Polycystic ovary syndrome: improving policies, awareness, and clinical care. Current Opinion in Endocrine and Metabolic Research 2020, 12, 112-118, doi:<http://dx.doi.org/10.1016/j.coemr.2020.04.007>. | **Wrong study design** |
| Garcia‐Beltran, C.; Malpique, R.; Carbonetto, B.; González‐Torres, P.; Henares, D.; Brotons, P.; Muñoz‐Almagro, C.; López‐Bermejo, A.; Zegher, F.; Ibáñez, L. Gut microbiota in adolescent girls with polycystic ovary syndrome: Effects of randomized treatments. Pediatric obesity 2021, 16, 1-11, doi:10.1111/ijpo.12734. | **Wrong outcome** |
| Gariani, K.; Hugon-Rodin, J.; Philippe, J.; Righini, M.; Blondon, M. Association between polycystic ovary syndrome and venous thromboembolism: A systematic review and meta-analysis. Thrombosis research 2020, 185, 102-108, doi:<https://dx.doi.org/10.1016/j.thromres.2019.11.019>. | **Wrong intervention** |
| Gateva, A.; Unfer, V.; Kamenov, Z. The use of inositol(s) isomers in the management of polycystic ovary syndrome: a comprehensive review. Gynecological endocrinology : the official journal of the International Society of Gynecological Endocrinology 2018, 34, 545-550, doi:<https://dx.doi.org/10.1080/09513590.2017.1421632>. | **Wrong study design** |
| Genazzani, A. Inositols: reflections on how to choose the appropriate one for PCOS. Gynecological endocrinology : the official journal of the International Society of Gynecological Endocrinology 2020, 36, 1045-1046, doi:<https://dx.doi.org/10.1080/09513590.2020.1846697> | **Wrong study design** |
| Glintborg, D.; Andersen, M. MANAGEMENT OF ENDOCRINE DISEASE: Morbidity in polycystic ovary syndrome. European journal of endocrinology 2017, 176, R53-R65. | **Wrong study design** |
| Goldrick, K.M.; Kostroun, K.E.; Mondshine, J.N.; Robinson, R.D.; Mankus, E.B.; Knudtson, J.F. METFORMIN SHOULD BE RECOMMENDED FOR MORE PATIENTS WITH PCOS BASED ON UPDATED GUIDELINES. Fertility & Sterility 2020, 114, e404-e405, doi:10.1016/j.fertnstert.2020.08.1184. | **Wrong publication type** |
| Gong, W.; Mi, Y.; Shi, Y. The effect of modified erchen decoction on reproductive endocrine functions and glucose metabolism in patients with phlegm-dampness polycystic ovary syndrome complicated with insulin resistance. International Journal of Clinical and Experimental Medicine 2020, 13, 5932-5940. | **Wrong intervention** |
| Gordon-Elliott, J.S.; Ernst, C.L.; Fersh, M.E.; Albertini, E.; Lusskin, S.I.; Altemus, M. The Hypothalamic-Pituitary-Gonadal Axis and Women's Mental Health: PCOS, Premenstrual Dysphoric Disorder, and Perimenopause. Psychiatric Times 2017, 34, 5-8. | **Wrong study design** |
| Goyal, M.; Dawood, A. Debates regarding lean patients with polycystic ovary syndrome: A narrative review. Journal of Human Reproductive Sciences 2017, 10, 154-161, doi:<http://dx.doi.org/10.4103/jhrs.JHRS_77_17>. | **Wrong study design** |
| Greenhill, C. PCOS: Metformin risk for offspring. Nature Reviews Endocrinology 2018, 14, 253-253, doi:10.1038/nrendo.2018.34. | **Wrong publication type** |
| Grindheim, S.; Ebbing, C.; Karlsen, H.O.; Skulstad, S.M.; Real, F.G.; Lonnebotn, M.; Lovvik, T.; Vanky, E.; Kessler, J. Metformin exposure, maternal PCOS status and fetal venous liver circulation: A randomized, placebo-controlled study. PloS one 2022, 17, e0262987, doi:<https://dx.doi.org/10.1371/journal.pone.0262987>. | **Wrong population** |
| Guan, C.; Zahid, S.; Minhas, A.S.; Ouyang, P.; Vaught, A.; Baker, V.L.; Michos, E.D. Polycystic ovary syndrome: a "risk-enhancing" factor for cardiovascular disease. Fertility & Sterility 2022, 117, 924-935, doi:10.1016/j.fertnstert.2022.03.009. | **Wrong study design** |
| Gunalan, E.; Yaba, A.; Yilmaz, B. The effect of nutrient supplementation in the management of polycystic ovary syndrome-associated metabolic dysfunctions: A critical review. Journal of the Turkish German Gynecology Association 2018, 19, 220-232, doi:<http://dx.doi.org/10.4274/jtgga.2018.0077>. | **Wrong study design** |
| Haas, J.; Bentov, Y. Should metformin be included in fertility treatment of PCOS patients? Medical hypotheses 2017, 100, 54-58, doi:10.1016/j.mehy.2017.01.012. | **Wrong study design** |
| Hakimi, O.; Cameron, L.-C. Effect of Exercise on Ovulation: A Systematic Review. Sports Medicine 2017, 47, 1555-1567, doi:10.1007/s40279-016-0669-8. | **Wrong population** |
| Hameed, L.; Farooq, A.D.; Qureshi, T. Analysis of Unani coded formulation on the hormonal parameters of patients with polycystic ovarian syndrome. Pakistan journal of pharmaceutical sciences 2021, 34, 899-907 | **Wrong intervention** |
| Hanem, L.G.E.; Stridsklev, S.; Juliusson, P.B.; Roelants, M.; Carlsen, S.M.; Odegard, R.; Vanky, E. Intrauterine metformin exposure influences offspring growth,-a 4-year follow-up of children born to mothers with polycystic ovary syndrome. Endocrine reviews 2017, 38. | **Wrong publication type** |
| Hanem, L.G.E.; Stridsklev, S.; Juliusson, P.B.; Salvesen, O.; Roelants, M.; Carlsen, S.M.; Odegard, R.; Vanky, E. Metformin Use in PCOS Pregnancies Increases the Risk of Offspring Overweight at 4 Years of Age: Follow-Up of Two RCTs. The Journal of clinical endocrinology and metabolism 2018, 103, 1612-1621, doi:<https://dx.doi.org/10.1210/jc.2017-02419>. | **Wrong outcome** |
| Hashim, H.A.; Shokeir, T.; Badawy, A. RETRACTED: Letrozole versus combined metformin and clomiphene citrate for ovulation induction in clomiphene-resistant women with polycystic ovary syndrome: a randomized controlled trial. Elsevier B.V.: New York, New York, 2020; Vol. 114, pp 667-667. | **Wrong publication type** |
| Hefny, M.; Mohammad, M.; Wafa, Y. Effect of N-acetylcysteine as an adjuvant to clomiphene citrate for induction of ovulation in patients with polycystic ovary syndrome. BJOG 2018, 125, 38, doi:<https://doi.org/10.1111/1471-0528.15132>. | **Wrong intervention** |
| Heidari, B.; Change, A.Y.; Lerman, L.O.; Lerman, A. Effect of metformin on microvascular endothelial function in polycystic ovary syndrome. Circulation 2018, 138. | **Wrong publication type** |
| Heidari, B.; Change, A.Y.; Lerman, L.O.; Lerman, A. Abstract 12145: Effect of Metformin on Microvascular Endothelial Function in Polycystic Ovary Syndrome. Circulation 2018, 138, A12145-A12145. | **Wrong publication type** |
| Helvaci, N.; Yildiz, B.O. Polycystic ovary syndrome and aging: Health implications after menopause. Maturitas 2020, 139, 12-19, doi:10.1016/j.maturitas.2020.05.013. | **Wrong study design** |
| Hibberd, R.; Raine‐Fenning, N.; Thornton, J. Re: Inositol treatment of anovulation in women with polycystic ovary syndrome: a meta-analysis of randomised trials. Wiley-Blackwell: Malden, Massachusetts, 2018; Vol. 125, pp 509-509. | **Wrong intervention** |
| Hjorth-Hansen, A.; Eggebo, T.; Salvesen, K.A.; Vanky, E.; Odegard, R. Head size and growth in utero and at birth in metformin exposed children born to mothers with PCOS-a randomized controlled trial. Endocrine reviews 2017, 38. | **Wrong publication type** |
| Hjorth-Hansen, A.; Salvesen, Ø.; Engen Hanem, L.G.; Eggebø, T.; Salvesen, K.Å.; Vanky, E.; Ødegård, R. Fetal Growth and Birth Anthropometrics in Metformin-Exposed Offspring Born to Mothers With PCOS. Journal of Clinical Endocrinology & Metabolism 2017, 10.1210/jc.2017-01191, N.PAG-N.PAG, doi:10.1210/jc.2017-01191 | **Wrong outcome** |
| Hjorth-Hansen, A.; Salvesen, O.; Engen Hanem, L.G.; Eggebo, T.; Salvesen, K.A.; Vanky, E.; Odegard, R. Fetal Growth and Birth Anthropometrics in Metformin-Exposed Offspring Born to Mothers With PCOS. The Journal of clinical endocrinology and metabolism 2018, 103, 740-747, doi:https://dx.doi.org/10.1210/jc.2017-01191. | **Wrong outcome** |
| Hu, A.C.; Chapman, L.W.; Mesinkovska, N.A. The efficacy and use of finasteride in women: a systematic review. International journal of dermatology 2019, 58, 759-776, doi:https://dx.doi.org/10.1111/ijd.14370. | **Wrong population** |
| Huang, C.D.O. Is metformin and spironolactone better than metformin alone for improving polycystic ovarian syndrome symptoms? Evidence-Based Practice 2019, 22, 31-32, doi:10.1097/EBP.0000000000000207. | **Wrong study design** |
| Huddleston, H.G.; Dokras, A. Diagnosis and Treatment of Polycystic Ovary Syndrome. JAMA: Journal of the American Medical Association 2022, 327, 274-275, doi:10.1001/jama.2021.23769. | **Wrong study design** |
| Ibanez, L.; Del Rio, L.; Diaz, M.; Sebastiani, G.; Pozo, O.J.; Lopez-Bermejo, A.; De Zegher, F.E. Ovulation rates after randomized interventions for polycystic ovary syndrome in adolescent girls. Endocrine reviews 2017, 38. | **Wrong publication type** |
| Ibanez, L.; Oberfield, S.E.; Witchel, S.; Auchus, R.J.; Chang, R.J.; Codner, E.; Dabadghao, P.; Darendeliler, F.; Elbarbary, N.S.; Gambineri, A., et al. An International Consortium Update: Pathophysiology, Diagnosis, and Treatment of Polycystic Ovarian Syndrome in Adolescence. Hormone research in paediatrics 2017, 88, 371-395, doi:https://dx.doi.org/10.1159/000479371. | **Wrong study design** |
| Ibáñez, L.; Zegher, F. Polycystic ovary syndrome in adolescent girls. Pediatric obesity 2020, 15, N.PAG-N.PAG, doi:10.1111/ijpo.12586. | **Wrong study design** |
| Iervolino, M.; Lepore, E.; Forte, G.; Laganà, A.S.; Buzzaccarini, G.; Unfer, V. Natural Molecules in the Management of Polycystic Ovary Syndrome (PCOS): An Analytical Review. Nutrients 2021, 13, 1677, doi:10.3390/nu13051677. | **Wrong study design** |
| Jam Ashkezari, S.; Namiranian, N.; Gholami, S.; Elahi, M.; Rahmanian, M. Evaluation of the metformin effects on Anti-Mullerian Hormone in women with polycystic ovarian syndrome: a double-blind randomized clinical trial. International Journal of Reproductive BioMedicine 2019, 17, 143, doi:https://doi.org/10.18502/8. | **Wrong publication type** |
| Jamilian, H.; Jamilian, M.; Foroozanfard, F.; Afshar Ebrahimi, F.; Bahmani, F.; Asemi, Z. Comparison of myo-inositol and metformin on mental health parameters and biomarkers of oxidative stress in women with polycystic ovary syndrome: a randomized, double-blind, placebo-controlled trial. Journal of Psychosomatic Obstetrics & Gynecology 2018, 39, 307-314, doi:10.1080/0167482X.2017.1383381. | **Wrong comparator** |
| Jamilian, M.; Farhat, P.; Foroozanfard, F.; Afshar Ebrahimi, F.; Aghadavod, E.; Bahmani, F.; Badehnoosh, B.; Jamilian, H.; Asemi, Z. Comparison of myo-inositol and metformin on clinical, metabolic and genetic parameters in polycystic ovary syndrome: a randomized controlled clinical trial. Clinical endocrinology 2017, 87, 194, doi:https://doi.org/10.1111/cen.13366. | **Wrong comparator** |
| Janati, S.; Behmanesh, M.A.; Najafzadehvarzi, H.; Kassani, A.; Athari, N.; Poormoosavi, S.M. Changes of Serum Level of Homocysteine and Oxidative Stress Markers by Metformin and Inositol in Infertile Women with Polycystic Ovary Syndrome: a Double Blind Randomized Clinical Trial Study. International Journal of Fertility and Sterility 2022, 16, 102, doi:https://doi.org/10.22074/IJFS.2021.530040.1125. | **Wrong comparator** |
| Janez, A.; Salamun, V.; Jensterle, M.; Bokal, E.V. Short-term intervention with liraglutide and metformin increased fertility potential in a subset of obese PCOS proceeding in vitro sterilisation. Diabetes 2017, 66, A561. | **Wrong publication type** |
| Javed, Z.; Papageorgiou, M.; Madden, L.A.; Rigby, A.S.; Kilpatrick, E.S.; Atkin, S.L.; Sathyapalan, T. The effects of empagliflozin vs metformin on endothelial microparticles in overweight/obese women with polycystic ovary syndrome. Endocrine Connections 2020, 9, 563-569, doi:http://dx.doi.org/10.1530/EC-20-0173. | **Wrong outcome** |
| Jazani, A.M.; Nazemiyeh, H.; Tansaz, M.; Bazargani, H.S.; Fazljou, S.M.B.; Azgomi, R.N.D.; Hamdi, K. Celery plus anise versus metformin for treatment of oligomenorrhea in polycystic ovary syndrome: a triple-blind, randomized clinical trial. Iranian Red Crescent Medical Journal 2018, 20, doi:https://doi.org/10.5812/ircmj.67181. | **Wrong comparator** |
| Jensterle, M.; Ferjan, S.; Janez, A. DPP4 inhibitor sitagliptin in combination with metformin prevent weight regain in obese women with pcos previously treated with liraglutide. Endocrine reviews 2017, 38. | **Wrong publication type** |
| Jensterle, M.; Goricar, K.; Janez, A. Add on DPP-4 inhibitor alogliptin alone or in combination with pioglitazone improved beta-cell function and insulin sensitivity in metformin treated PCOS. Endocrine research 2017, https://doi.org/10.1080/07435800.2017.1294602, 1, doi:https://doi.org/10.1080/07435800.2017.1294602. | **Wrong intervention** |
| Jensterle, M.; Salamun, V.; Bokal, E.V.; Janez, A. Short-term intervention with liraglutide and metformin increased fertility potential in a subset of obese women with PCOS proceeding in vitro fertilization. Endocrine reviews 2017, 38. | **Wrong publication type** |
| Jiang, J.; Gao, S.; Zhang, Y. Therapeutic effects of dimethyldiguanide combined with clomifene citrate in the treatment of polycystic ovary syndrome. Revista da Associacao Medica Brasileira (1992) 2019, 65, 1144-1150, doi:https://dx.doi.org/10.1590/1806-9282.65.9.1144. | **Wrong intervention** |
| Jiang, Q.; Shi, Y. Effect of orlistat on obese women with polycystic ovary syndrome. Journal of Bio-X Research 2018, 1, 128-131, doi:https://dx.doi.org/10.1097/JBR.0000000000000017. | **Wrong study design** |
| Jiang, S.; Tang, T.; Sheng, Y.; Li, R.; Xu, H. The Effects of Letrozole and Metformin Combined with Targeted Nursing Care on Ovarian Function, LH, and FSH in Infertile Patients with Polycystic Ovary Syndrome. Journal of healthcare engineering 2022, 2022, 3712166, doi:https://dx.doi.org/10.1155/2022/3712166. | **Wrong study design** |
| Jin, P.; Xie, Y. Treatment strategies for women with polycystic ovary syndrome. Gynecological endocrinology : the official journal of the International Society of Gynecological Endocrinology 2018, 34, 272-277, doi:https://dx.doi.org/10.1080/09513590.2017.1395841. | **Wrong study design** |
| Kachhawa, G.; Senthil Kumar, K.V.; Kulshrestha, V.; Khadgawat, R.; Mahey, R.; Bhatla, N. Efficacy of myo-inositol and d-chiro-inositol combination on menstrual cycle regulation and improving insulin resistance in young women with polycystic ovary syndrome: a randomized open-label study. International journal of gynaecology and obstetrics 2021, https://doi.org/10.1002/ijgo.13971, doi:https://doi.org/10.1002/ijgo.13971. | **Wrong comparator** |
| Kamboj, M.K.; Bonny, A.E. Polycystic ovary syndrome in adolescence: Diagnostic and therapeutic strategies. Translational Pediatrics 2017, 6, 248-255, doi:http://dx.doi.org/10.21037/tp.2017.09.11. | **Wrong study design** |
| Kamenov, Z.; Gateva, A. Inositols in PCOS. Molecules (Basel, Switzerland) 2020, 25, doi:https://dx.doi.org/10.3390/molecules25235566. | **Wrong study design** |
| Kancherla, H.; Konduri, G.; Gelly, R.B.; Tadikonda, R.R. Diagnosis and Treatment of Polycystic Ovary Syndrome (PCOS)-A Comparative Review. International Journal of Pharmaceutical Sciences Review and Research 2022, 73, 107-113, doi:https://dx.doi.org/10.47583/ijpsrr.2022.v73i01.018. | **Wrong study design** |
| Kataoka, J.; Tassone, E.C.; Misso, M.; Joham, A.E.; Stener-Victorin, E.; Teede, H.; Moran, L.J. Weight Management Interventions in Women with and without PCOS: A Systematic Review. Nutrients 2017, 9, 996, doi:10.3390/nu9090996. | **Wrong intervention** |
| Kaur, M.D.O.; Silva, B.D.O.; Retailliau, L.D.O. Which treatments are effective at achieving weight loss among overweight or obese reproductive age women with polycystic ovary syndrome? Evidence-Based Practice 2022, 25, 35-36, doi:10.1097/EBP.0000000000001431. | **Wrong study design** |
| Khan, A.A.; Begum, W. Efficacy of Darchini in the management of polycystic ovarian syndrome: A randomized clinical study. Journal of Herbal Medicine 2019, 15, 100249, doi:http://dx.doi.org/10.1016/j.hermed.2018.11.005. | **Wrong comparator** |
| Khan, L. Polycystic Ovarian Syndrome in Adolescents: Keys to Diagnosis and Management. Pediatric annals 2021, 50, e272-e275, doi:10.3928/19382359-20210622-01. | **Wrong study design** |
| Kialka, M.; Galuszka-Bednarczyk, A.; Wajda, A.; Czekanska, P.; Zdzierak, B.; Mrozinska, S.; Janeczko, M.; Milewicz, T. Metformin and changes in serum lipid profile in lean patients with polycystic ovary syndrome. Przeglad lekarski 2017, 74, 144-146. | **Fulltext not obtainable** |
| Kim, H.H. Androgens, estrogens, and cardiovascular disease: considerations for women with polycystic ovary syndrome. Fertility & Sterility 2019, 112, 478-479, doi:10.1016/j.fertnstert.2019.06.025. | **Wrong study design** |
| Kim, Y.; Yoon, S.; Ku, S.; Lee, S.; Shin, J.; Kim, T.; Hur, J. Effect of oral contraceptives over 1-year on change in body composition profiles of women with polycystic ovary syndrome: a cohort study. Fertility & Sterility 2017, 108, e248-e248, doi:10.1016/j.fertnstert.2017.07.745. | **Wrong study design** |
| Kini, S.; Ramalingam, M. Hirsutism. Obstetrics, Gynaecology and Reproductive Medicine 2018, 28, 129-135, doi:http://dx.doi.org/10.1016/j.ogrm.2018.03.004. | **Wrong study design** |
| Kolivand, M.; Keramat, A.; Khosravi, A. The Effect of Herbal Teas on Management of Polycystic Ovary Syndrome: A Systematic Review. Journal of Midwifery & Reproductive Health 2017, 5, 1098-1106, doi:10.22038/JMRH.2017.9368. | **Wrong intervention** |
| Kostopoulou, E.; Anagnostis, P.; Bosdou, J.K.; Spiliotis, B.E.; Goulis, D.G. Polycystic ovary Syndrome in Adolescents: Pitfalls in Diagnosis and Management. Current obesity reports 2020, 9, 193-203, doi:https://dx.doi.org/10.1007/s13679-020-00388-9. | **Wrong study design** |
| Kriedt, K.J.; Alchami, A.; Davies, M.C. PCOS: diagnosis and management of related infertility. Obstetrics, Gynaecology & Reproductive Medicine 2019, 29, 1-5, doi:10.1016/j.ogrm.2018.12.001. | **Wrong study design** |
| Krysiak, R.; Gilowska, M.; Okopien, B. The effect of oral contraception on cardiometabolic risk factors in women with elevated androgen levels. Pharmacological reports : PR 2017, 69, 45-49, doi:https://dx.doi.org/10.1016/j.pharep.2016.09.013. | **Fulltext not obtainable** |
| Kulkarni, D.; Pai, S.; Ayyar, V.; Bantwal, G.; George, B.; Appaiah, S. Effect of metformin and Vitamin E compared to lifestyle modification on AST/Platelet ratio in PCOS patients with associated NASH. Indian Journal of Endocrinology and Metabolism 2022, 26, S36. | **Wrong publication type** |
| Kwon, C.-Y.; Lee, B.; Park, K.S. Oriental herbal medicine and moxibustion for polycystic ovary syndrome: A meta-analysis. Medicine 2018, 97, e12942-e12942, doi:10.1097/MD.0000000000012942. | **Wrong intervention** |
| Lagana, A.S.; Rossetti, P.; Sapia, F.; Chiofalo, B.; Buscema, M.; Valenti, G.; Rapisarda, A.M.C.; Vitale, S.G. Evidence-based and patient-oriented inositol treatment in polycystic ovary syndrome: Changing the perspective of the disease. International Journal of Endocrinology and Metabolism 2017, 15, e43695, doi:http://dx.doi.org/10.5812/ijem.43695. | **Wrong study design** |
| Laganà, A.S.; Vitagliano, A.; Noventa, M.; Ambrosini, G.; D’Anna, R.; D'Anna, R. Myo-inositol supplementation reduces the amount of gonadotropins and length of ovarian stimulation in women undergoing IVF: a systematic review and meta-analysis of randomized controlled trials. Archives of Gynecology & Obstetrics 2018, 298, 675-684, doi:10.1007/s00404-018-4861-y. | **Wrong intervention** |
| Lamos, E.M.; Malek, R.; Davis, S.N. GLP-1 receptor agonists in the treatment of polycystic ovary syndrome. Expert review of clinical pharmacology 2017, 10, 401-408, doi:https://dx.doi.org/10.1080/17512433.2017.1292125. | **Wrong study design** |
| Lazaridou, S.; Dinas, K.; Tziomalos, K. Prevalence, pathogenesis and management of prediabetes and type 2 diabetes mellitus in patients with polycystic ovary syndrome. Hormones (Athens, Greece) 2017, 16, 373-380, doi:https://dx.doi.org/10.14310/horm.2002.1757. | **Wrong study design** |
| Le, T.N.; Wickham, E.P.R.; Nestler, J.E. Insulin sensitizers in adolescents with polycystic ovary syndrome. Minerva pediatrica 2017, 69, 434-443, doi:https://dx.doi.org/10.23736/S0026-4946.17.04976-3. | **Wrong study design** |
| Lepine, S.; Jo, J.; Metwally, M.; Cheong, Y.C. Ovarian surgery for symptom relief in women with polycystic ovary syndrome. The Cochrane database of systematic reviews 2017, 11, CD009526, doi:https://dx.doi.org/10.1002/14651858.CD009526.pub2. | **Wrong comparator** |
| Levin, G.; Rottenstreich, A. Inositol for women with polycystic ovary syndrome-possibly just better than placebo. Wiley-Blackwell: Malden, Massachusetts, 2019; Vol. 98, pp 262-262. | **Wrong study design** |
| Li, M.F.; Zhou, X.M.; Li, X.L. The Effect of Berberine on Polycystic Ovary Syndrome Patients with Insulin Resistance (PCOS-IR): A Meta-Analysis and Systematic Review. Evidence-based Complementary and Alternative Medicine 2018, 2018, 2532935, doi:http://dx.doi.org/10.1155/2018/2532935. | **Wrong comparator** |
| Li, R.; Zheng, S.; Mai, T.; Xue, J.; Zhang, Y. Comparison of the effects of metformin and exenatide on pregnancy rate and outcomes in overweight or obese pcos women. Diabetes 2020, 69, doi:https://doi.org/10.2337/db20-1341-P. | **Wrong outcome** |
| Li, S.; Wang, Y.; Cai, J.; Liu, W.; Yin, H.; Tao, T. Lifestyle intervention, metformin, and acarbose treatments differentially impact liver fat content, serum lipids, and hormone profiles in obese polycystic ovary syndrome patients with impaired glucose tolerance. Diabetes 2020, 69, doi:https://doi.org/10.2337/db20-2013-P. | **Wrong outcome** |
| Li, X.; Celotto, S.; Pizzol, D.; Gasevic, D.; Ji, M.-M.; Barnini, T.; Solmi, M.; Stubbs, B.; Smith, L.; Lopez Sanchez, G.F., et al. Metformin and health outcomes: An umbrella review of systematic reviews with meta-analyses. European journal of clinical investigation 2021, 51, e13536, doi:https://dx.doi.org/10.1111/eci.13536. | **Wrong population** |
| Li, Y.; Chen, C.; Ma, Y.; Xiao, J.; Luo, G.; Li, Y.; Wu, D. Multi-system reproductive metabolic disorder: significance for the pathogenesis and therapy of polycystic ovary syndrome (PCOS). Life sciences 2019, 228, 167-175, doi:https://dx.doi.org/10.1016/j.lfs.2019.04.046. | **Wrong study design** |
| Lim, C.E.D.; Ng, R.W.C.; Cheng, N.C.L.; Zhang, G.S.; Chen, H. Acupuncture for polycystic ovarian syndrome. The Cochrane database of systematic reviews 2019, 7, CD007689, doi:https://dx.doi.org/10.1002/14651858.CD007689.pub4. | **Wrong intervention** |
| Lim, S.S.; Hutchison, S.K.; Van Ryswyk, E.; Norman, R.J.; Teede, H.J.; Moran, L.J. Lifestyle changes in women with polycystic ovary syndrome. The Cochrane database of systematic reviews 2019, 3, CD007506, doi:https://dx.doi.org/10.1002/14651858.CD007506.pub4. | **Wrong intervention** |
| Lin, L.; Wang, F.; Chen, M.X.; Mo, Z.W.; Fang, T.Y.; Quan, H.B. [Pulse administration of gonadotropin-releasing hormone combined with metformin for fertility in a non-obese woman with polycystic ovary syndrome]. Zhonghua nei ke za zhi 2019, 58, 531-533, doi:https://dx.doi.org/10.3760/cma.j.issn.0578-1426.2019.07.009. | **Fulltext not obtainable** |
| Lin, W.; Feng, J.; Zhou, H.; Chen, X.; Diao, W.; Ma, P. Therapeutic efficacy of clomiphene citrate combined with metformin in patients with polycystic ovary syndrome. Journal of Clinical Pharmacy & Therapeutics 2022, 47, 321-329, doi:10.1111/jcpt.13561. | **Wrong outcome** |
| Liu, C.; Feng, G.; Huang, W.; Wang, Q.; Yang, S.; Tan, J.; Fu, J.; Liu, D. Comparison of clomiphene citrate and letrozole for ovulation induction in women with polycystic ovary syndrome: a prospective randomized trial. Gynecological endocrinology : the official journal of the International Society of Gynecological Endocrinology 2017, 33, 872-876, doi:https://dx.doi.org/10.1080/09513590.2017.1332174. | **Wrong intervention** |
| Liu, R.; Li, M.; Wang, P.; Yu, M.; Wang, Z.; Zhang, G.Z. Preventive online and offline health management intervention in polycystic ovary syndrome. World Journal of Clinical Cases 2022, 10, 3060-3068, doi:https://dx.doi.org/10.12998/wjcc.v10.i10.3060. | **Wrong intervention** |
| Liu, R.-B.; Liu, Y.; Lv, L.-Q.; Xiao, W.; Gong, C.; Yue, J.-X. Effects of Metformin Treatment on Soluble Leptin Receptor Levels in Women with Polycystic Ovary Syndrome. Current medical science 2019, 39, 609-614, doi:https://dx.doi.org/10.1007/s11596-019-2081-8. | **Wrong study design** |
| Livadas, S.; Anagnostis, P.; Bosdou, J.K.; Bantouna, D.; Paparodis, R. Polycystic ovary syndrome and type 2 diabetes mellitus: A state-ofthe- art review. World Journal of Diabetes 2022, 13, 5-26, doi:https://dx.doi.org/10.4239/wjd.v13.i1.5. | **Wrong study design** |
| Lovvik, T.S.; Carlsen, S.M.; Salvesen, O.; Steffensen, B.; Bixo, M.; Gomez-Real, F.; Lonnebotn, M.; Hestvold, K.V.; Zabielska, R.; Hirschberg, A.L., et al. Use of metformin to treat pregnant women with polycystic ovary syndrome (PregMet2): a randomised, double-blind, placebo-controlled trial. The lancet. Diabetes & endocrinology 2019, 7, 256-266, doi:https://dx.doi.org/10.1016/S2213-8587(19)30002-6. | **Wrong population** |
| Luque-Ramirez, M.; Ortiz-Flores, A.E.; Nattero-Chavez, L.; Escobar-Morreale, H.F. A safety evaluation of current medications for adult women with the polycystic ovarian syndrome not pursuing pregnancy. Expert opinion on drug safety 2020, 19, 1559-1576, doi:https://dx.doi.org/10.1080/14740338.2020.1839409. | **Wrong study design** |
| Macut, D.; Bjekić-Macut, J.; Rahelić, D.; Doknić, M. Insulin and the polycystic ovary syndrome. Diabetes Research & Clinical Practice 2017, 130, 163-170, doi:10.1016/j.diabres.2017.06.011. | **Wrong study design** |
| Magzoub, R.; Kheirelseid, E.A.H.; Perks, C.; Lewis, S. Does metformin improve reproduction outcomes for non-obese, infertile women with polycystic ovary syndrome? Meta-analysis and systematic review. European Journal of Obstetrics & Gynecology & Reproductive Biology 2022, 269, 38-62, doi:10.1016/j.ejogrb.2022.01.025. | **Wrong outcome** |
| Mahmood, S.; Answer, S. Metformin and pioglitazone comparison for ovulation induction in PCOS. BJOG 2021, 128, 230, doi:https://doi.org/10.1111/1471-0528.18-16715. | **Wrong publication type** |
| Makaya, T.; Basu, S.; Poole, R. Management of teenagers with polycystic ovarian syndrome. Paediatrics & Child Health 2019, 29, 303-308, doi:10.1016/j.paed.2019.04.004. | **Wrong study design** |
| Maleki, V.; Izadi, A.; Farsad-Naeimi, A.; Alizadeh, M. Chromium supplementation does not improve weight loss or metabolic and hormonal variables in patients with polycystic ovary syndrome: A systematic review. Nutrition Research 2018, 56, 1-10, doi:10.1016/j.nutres.2018.04.003. | **Wrong intervention** |
| Malhotra, N.; Mahey, R.; Agarwal, A.; Rajasekaran, K.; Gupta, M. Short term effects of metformin, myo-inositol or combination on metabolic and endocrine profile of infertile women with polycystic ovarian syndrome (PCOS). Human reproduction (Oxford, England) 2019, 34, i421. | **Wrong publication type** |
| Manzoor, S.; Ganie, M.A.; Amin, S.; Shah, Z.A.; Bhat, I.A.; Yousuf, S.D.; Jeelani, H.; Kawa, I.A.; Fatima, Q.; Rashid, F. Oral contraceptive use increases risk of inflammatory and coagulatory disorders in women with Polycystic Ovarian Syndrome: An observational study. Scientific reports 2019, 9, 10182, doi:https://dx.doi.org/10.1038/s41598-019-46644-4. | **Wrong study design** |
| Manzoor, S.; Ganie, M.A.; Majid, S.; Shabir, I.; Kawa, I.A.; Fatima, Q.; Jeelani, H.; Yousuf, S.D.; Rashid, F. Analysis of Intrinsic and Extrinsic Coagulation Pathway Factors in OCP Treated PCOS Women. Indian Journal of Clinical Biochemistry 2021, 36, 278-287, doi:http://dx.doi.org/10.1007/s12291-020-00901-w. | **Wrong study design** |
| Marciniak, A.; Lejman-Larysz, K.; Nawrocka-Rutkowska, J.; Brodowska, A.; Songin, D. [Polycystic ovary syndrome - current state of knowledge]. Zespol policystycznych jajnikow - aktualny stan wiedzy. 2018, 44, 296-301. | **Wrong language** |
| Markowicz-Piasecka, M.; Huttunen, K.M.; Mateusiak, L.; Mikiciuk-Olasik, E.; Sikora, J. Is Metformin a Perfect Drug? Updates in Pharmacokinetics and Pharmacodynamics. Current pharmaceutical design 2017, 23, 2532-2550, doi:https://dx.doi.org/10.2174/1381612822666161201152941. | **Wrong study design** |
| Mascarenhas, M.; Balen, A.H. Treatment update for anovulation and subfertility in polycystic ovary syndrome. Current Opinion in Endocrine and Metabolic Research 2020, 12, 53-58, doi:http://dx.doi.org/10.1016/j.coemr.2020.03.003. | **Wrong study design** |
| Matrood, R.H.; Abdulhussain, A.S. The added effect of cabergoline to metformin on serum hormones and rate and regularity of menstruation in women with polycystic ovary syndrome. International Journal of Research in Pharmaceutical Sciences 2018, 9, 243-248, doi:http://dx.doi.org/10.26452/ijrps.v9i1.1255. | **Fulltext not obtainable** |
| Maysara, A.M.; Nassar, A.T.; Jubran, H.K. The effect of correction of serum level of vitamin D on hyperandrogenism in women with polycystic ovary syndrome and hypovitaminosis D. Clinical and experimental obstetrics & gynecology 2020, 47, 272, doi:https://doi.org/10.31083/j.ceog.2020.02.5248. | **Wrong comparator** |
| McLean, W. Reviews of medical journal articles. Australian Journal of Herbal & Naturopathic Medicine 2019, 31, 110-116. | **Wrong study design** |
| Mendoza, N.; Perez, L.; Simoncini, T.; Genazzani, A. Inositol supplementation in women with polycystic ovary syndrome undergoing intracytoplasmic sperm injection: a systematic review and meta-analysis of randomized controlled trials. Reproductive biomedicine online 2017, 35, 529-535, doi:https://dx.doi.org/10.1016/j.rbmo.2017.07.005. | **Wrong intervention** |
| Meng, J.; Zhu, Y. Efficacy of simvastatin plus metformin for polycystic ovary syndrome: A meta-analysis of randomized controlled trials. European Journal of Obstetrics & Gynecology & Reproductive Biology 2020, 255, 19-24, doi:10.1016/j.ejogrb.2020.11.070. | **Wrong comparator** |
| Meng, J.; Zhu, Y. Efficacy of simvastatin plus metformin for polycystic ovary syndrome: a meta-analysis of randomized controlled trials. European journal of obstetrics and gynecology and reproductive biology 2021, 257, 19, doi:https://doi.org/10.1016/j.ejogrb.2020.11.070. | **Wrong comparator** |
| Merviel, P.; James, P.; Bouée, S.; Le Guillou, M.; Rince, C.; Nachtergaele, C.; Kerlan, V. Impact of myo-inositol treatment in women with polycystic ovary syndrome in assisted reproductive technologies. Reproductive health 2021, 18, 1-8, doi:10.1186/s12978-021-01073-3. | **Wrong study design** |
| Miankouhi, T.A.; Azadi, M. Evaluation of medical and traditional treatments on the fertility of women with polycystic ovary syndrome. Journal of Reproduction and Infertility 2018, 19, 115-116. | **Wrong study design** |
| Miao, K.; Zhou, H. Effect of statins combined or not combined with metformin on polycystic ovary syndrome: A systematic review and meta‐analysis. Journal of Obstetrics & Gynaecology Research 2022, 48, 1806-1815, doi:10.1111/jog.15301. | **Wrong intervention** |
| Mimoto, M.S.; Oyler, J.L.; Davis, A.M. Evaluation and Treatment of Hirsutism in Premenopausal Women. JAMA: Journal of the American Medical Association 2018, 319, 1613-1614, doi:10.1001/jama.2018.2611. | **Wrong population** |
| Mohammed, S.B.; Nayak, B.S. Polycystic ovarian syndrome trend in a nutshell. International Journal of Women's Health and Reproduction Sciences 2017, 5, 153-157, doi:http://dx.doi.org/10.15296/ijwhr.2017.28. | **Wrong study design** |
| Mohsin, R.; Saeed, A.; Baig, M.M.; Khan, M. Role of letrozole and metformin vs letrozole alone in ovulation induction in patients of polycystic ovarian syndrome. Pakistan Journal of Medical and Health Sciences 2019, 13, 350-352. | **Wrong comparator** |
| Mokaberinejad, R.; Rampisheh, Z.; Aliasl, J.; Akhtari, E. The comparison of fennel infusion plus dry cupping versus metformin in management of oligomenorrhoea in patients with polycystic ovary syndrome: a randomised clinical trial. Journal of Obstetrics & Gynaecology 2019, 39, 652-658, doi:10.1080/01443615.2018.1541232. | **Wrong comparator** |
| Molin, J.; Vanky, E.; Løvvik, T.S.; Dehlin, E.; Bixo, M. Gestational weight gain, appetite regulating hormones, and metformin treatment in polycystic ovary syndrome: A longitudinal, placebo‐controlled study. BJOG: An International Journal of Obstetrics & Gynaecology 2022, 129, 1112-1121, doi:10.1111/1471-0528.17042. | **Wrong population** |
| Monastra, G.; Unfer, V.; Harrath, A.H.; Bizzarri, M. Combining treatment with myo-inositol and D-chiro-inositol (40:1) is effective in restoring ovary function and metabolic balance in PCOS patients. Gynecological endocrinology : the official journal of the International Society of Gynecological Endocrinology 2017, 33, 1-9, doi:https://dx.doi.org/10.1080/09513590.2016.1247797. | **Wrong study design** |
| Monastra, G.; Vucenik, I.; Harrath, A.H.; Alwasel, S.H.; Kamenov, Z.A.; Lagana, A.S.; Monti, N.; Fedeli, V.; Bizzarri, M. PCOS and Inositols: Controversial Results and Necessary Clarifications. Basic Differences Between D-Chiro and Myo-Inositol. Frontiers in endocrinology 2021, 12, 660381, doi:https://dx.doi.org/10.3389/fendo.2021.660381. | **Wrong study design** |
| Moramezi, F.; Ghanbarzadeh, R.; Nikbakht, R. VP07.11: Comparison of the efficacy of metformin and inofolic in ovulation induction in patients with resistant polycystic ovarian syndrome. Ultrasound in Obstetrics & Gynecology 2021, 58, 127-127, doi:10.1002/uog.24142. | **Wrong publication type** |
| Morgante, G.; Massaro, M.G.; Di Sabatino, A.; Cappelli, V.; De Leo, V. Therapeutic approach for metabolic disorders and infertility in women with PCOS. Gynecological endocrinology : the official journal of the International Society of Gynecological Endocrinology 2018, 34, 4-9, doi:https://dx.doi.org/10.1080/09513590.2017.1370644. | **Wrong study design** |
| Morotti, E.; Giovanni Artini, P.; Persico, N.; Battaglia, C. Metformin metabolic and vascular effects in overweight/moderately obese hyperinsulinemic PCOS patients treated with contraceptive vaginal ring: a pilot study. Gynecological endocrinology : the official journal of the International Society of Gynecological Endocrinology 2019, 35, 854-861, doi:https://dx.doi.org/10.1080/09513590.2019.1613361. | **Wrong comparator** |
| Morsy, A.A.; Sabri, N.A.; Mourad, A.M.; Mojahed, E.M.; Shawki, M.A. Randomized controlled open‐label study of the effect of vitamin E supplementation on fertility in clomiphene citrate‐resistant polycystic ovary syndrome. Journal of Obstetrics & Gynaecology Research 2020, 46, 2375-2382, doi:10.1111/jog.14467. | **Wrong comparator** |
| Mueck, A.O.; global, A.g. Treatment of hyperandrogenism in women with ethinylestradiol and cyproteroneacetate. The European journal of contraception & reproductive health care : the official journal of the European Society of Contraception 2017, 22, 170-171, doi:https://dx.doi.org/10.1080/13625187.2017.1328170. | **Wrong study design** |
| Muharam, R.; Srilestari, A.; Mihardja, H.; Callestya, L.J.; Harzif, A.K. Combination of electroacupuncture and pharmacological treatment improves insulin resistance in women with polycystic ovary syndrome: double-blind randomized clinical trial. International Journal of Reproductive BioMedicine 2022, 20, 289, doi:https://doi.org/10.18502/ijrm.v20i4.10900. | **Wrong intervention** |
| Muhas, C.; Nishad, K.M.; Ummunnoora, K.P.; Jushna, K.; Saheera, K.V.; Dilsha, K.P. Polycystic ovary syndrome (PCOS)-an overview. International Journal of Current Pharmaceutical Research 2018, 10, 5-9, doi:http://dx.doi.org/10.22159/ijcpr.2018v10i6.30969. | **Wrong study design** |
| Naderpoor, N.; Gibson-Helm, M.; Shorakae, S.; Joham, A.; Bateson. Polycystic ovary syndrome Optimal management in general practice. Medicine Today 2017, 18, 55-59. | **Wrong study design** |
| Nas, K.; Tuu, L. A comparative study between myo-inositol and metformin in the treatment of insulin-resistant women. European review for medical and pharmacological sciences 2017, 21, 77-82. | **Wrong study design** |
| Nazirudeen, R.; Natarajan, V.; Jayaraman, S.; Subbiah, S. A randomized control trial comparing myoinositol based therapy in combination with metformin versus metformin monotherapy on the clinical and hormonal parameters in obese reproductive age women with polycystic ovarian syndrome. Indian Journal of Endocrinology and Metabolism 2022, 26, S16. | **Wrong publication type** |
| Nehra, J.; Kaushal, J.; Singhal, S.R.; Ghalaut, V. Effect of myoinositol versus metformin on biochemical profile in polycystic ovarian syndrome in women. British journal of clinical pharmacology 2019, 85, 1654, doi:https://doi.org/10.1111/bcp.13937. | **Wrong comparator** |
| Nehra, J.; Kaushal, J.; Singhal, S.R.; Ghalaut, V.S. A comparative study of myo inositol versus metformin on biochemical profile in polycystic ovarian syndrome in women. International Journal of Pharmaceutical Sciences and Research 2017, 8, 1664, doi:https://doi.org/10.13040/IJPSR.0975-8232.8(4).1664-70. | **Wrong comparator** |
| Nehra, J.; Kaushal, J.; Singhal, S.R.; Ghalaut, V.S. Comparision of myo-inositol versus metformin on anthropometric parameters in polycystic ovarian syndrome in women. International Journal of Pharmacy and Pharmaceutical Sciences 2017, 9, 144-148, doi:http://dx.doi.org/10.22159/ijpps.2017v9i4.16359. | **Wrong comparator** |
| Nemati, M.; Nemati, S.; Taheri, A.M.; Heidari, B. Comparison of metformin and N-acetyl cysteine, as an adjuvant to clomiphene citrate, in clomiphene-resistant women with polycystic ovary syndrome. Journal of gynecology obstetrics and human reproduction 2017, 46, 579-585, doi:https://dx.doi.org/10.1016/j.jogoh.2017.07.004. | **Wrong comparator** |
| Nikolakis, G.; Kyrgidis, A.; Zouboulis, C.C. Antiandrogens as a therapeutic option for hidradenitis suppurativa/ acne inversa. Experimental dermatology 2019, 28, 14, doi:https://doi.org/10.1111/exd.13893. | **Wrong study design** |
| Ning, D.; Rensong, Y.; Lizhen, W.; Hongjing, Y.; Ding, N.; Yue, R.; Wang, L.; Yang, H. Chinese herbal medicine on treating obese women with polycystic ovary syndrome: A systematic review and meta-analysis protocol. Medicine 2020, 99, 1-5, doi:10.1097/MD.0000000000022982. | **Wrong intervention** |
| Noreen, H.; Un Nisa Rab Nawaz, Z.; Khanum, W.; Syed, S.; Saleem, H.; Tanveer, I. Effectiveness of myoinositol versus metformin on biochemical profile of women with PCOS. BJOG 2021, 128, 236, doi:https://doi.org/10.1111/1471-0528.18-16715. | **Wrong publication type** |
| Notaro, A.L.G.; Neto, F.T.L. The use of metformin in women with polycystic ovary syndrome: an updated review. Journal of assisted reproduction and genetics 2022, 39, 573-579, doi:https://dx.doi.org/10.1007/s10815-022-02429-9. | **Wrong study design** |
| Nylander, M.; Frossing, S.; Clausen, H.V.; Kistorp, C.; Faber, J.; Skouby, S.O. Effects of liraglutide on ovarian dysfunction in polycystic ovary syndrome: a randomized clinical trial. Reproductive biomedicine online 2017, 35, 121, doi:https://doi.org/10.1016/j.rbmo.2017.03.023. | **Wrong outcome** |
| Oliveira, F.R.; Mamede, M.; Bizzi, M.F.; Rocha, A.L.L.; Ferreira, C.N.; Gomes, K.B.; Candido, A.L.; Reis, F.M. Effects of Short Term Metformin Treatment on Brown Adipose Tissue Activity and Plasma Irisin Levels in Women with Polycystic Ovary Syndrome: A Randomized Controlled Trial. Hormone and metabolic research = Hormon- und Stoffwechselforschung = Hormones et metabolisme 2020, 52, 718-723, doi:https://dx.doi.org/10.1055/a-1157-0615. | **Wrong outcome** |
| Ortiz-Flores, A.E.; Luque-Ramirez, M.; Escobar-Morreale, H.F. Pharmacotherapeutic management of comorbid polycystic ovary syndrome and diabetes. Expert opinion on pharmacotherapy 2018, 19, 1915-1926, doi:https://dx.doi.org/10.1080/14656566.2018.1528231. | **Wrong study design** |
| Otto-Buczkowska, E.; Grzyb, K.; Jainta, N. Polycystic ovary syndrome (PCOS) and the accompanying disorders of glucose homeostasis among girls at the time of puberty. Pediatric endocrinology, diabetes, and metabolism 2018, 24, 40-44, doi:https://dx.doi.org/10.18544/PEDM-24.01.0101. | **Wrong study design** |
| Ozay, A.C.; Emekci Ozay, O.; Okyay, R.E.; Gulekli, B. The effect of myoinositol on ovarian blood flows in women with polycystic ovary syndrome. Gynecological endocrinology : the official journal of the International Society of Gynecological Endocrinology 2019, 35, 237-241, doi:<https://dx.doi.org/10.1080/09513590.2018.1520827>. | **Wrong study design** |
| Pal Singh Kochar, I.; Ramachandran, S.; Sethi, A. Metformin in Adolescent PCOS: The Way Forward. Pediatric endocrinology reviews : PER 2017, 15, 142-146, doi:https://dx.doi.org/10.17458/per.vol15.2017.prs.metforminadolescentpcos | **Wrong study design** |
| Pani, A.; Gironi, I.; Di Vieste, G.; Mion, E.; Bertuzzi, F.; Pintaudi, B. From Prediabetes to Type 2 Diabetes Mellitus in Women with Polycystic Ovary Syndrome: Lifestyle and Pharmacological Management. International Journal of Endocrinology 2020, 10.1155/2020/6276187, 1-10, doi:10.1155/2020/6276187. | **Wrong study design** |
| Papaetis, G.S.; Filippou, P.K.; Constantinidou, K.G.; Stylianou, C.S. Liraglutide: New Perspectives for the Treatment of Polycystic Ovary Syndrome. Clinical drug investigation 2020, 40, 695-713, doi:<https://dx.doi.org/10.1007/s40261-020-00942-2>. | **Wrong study design** |
| Pasquali, R. Contemporary approaches to the management of polycystic ovary syndrome. Therapeutic Advances in Endocrinology and Metabolism 2018, 9, 123-134, doi:http://dx.doi.org/10.1177/2042018818756790. | **Wrong study design** |
| Patel, S. Polycystic ovary syndrome (PCOS), an inflammatory, systemic, lifestyle endocrinopathy. The Journal of steroid biochemistry and molecular biology 2018, 182, 27-36, doi:https://dx.doi.org/10.1016/j.jsbmb.2018.04.008. | **Wrong study design** |
| Pedersen, A.J.T.; Stage, T.B.; Glintborg, D.; Andersen, M.; Christensen, M.M.H. The Pharmacogenetics of Metformin in Women with Polycystic Ovary Syndrome: a Randomized Trial (in press). Basic & clinical pharmacology & toxicology 2017. | **Wrong study design** |
| Pedersen, A.J.T.; Stage, T.B.; Glintborg, D.; Andersen, M.; Christensen, M.M.H. The Pharmacogenetics of Metformin in Women with Polycystic Ovary Syndrome: a Randomized Trial. Basic & clinical pharmacology & toxicology 2018, 122, 239, doi:https://doi.org/10.1111/bcpt.12874. | **Wrong study design** |
| Perichart-Perera, O.; Mier-Cabrera, J.; Flores-Robles, C.M.; Martinez-Cruz, N.; Arce-Sanchez, L.; Alvarado-Maldonado, I.N.; Montoya-Estrada, A.; Romo-Yanez, J.; Rodriguez-Cano, A.M.; Estrada-Gutierrez, G., et al. Intensive Medical Nutrition Therapy Alone or with Added Metformin to Prevent Gestational Diabetes Mellitus among High-Risk Mexican Women: A Randomized Clinical Trial. Nutrients 2021, 14, doi:https://dx.doi.org/10.3390/nu14010062. | **Wrong population** |
| Perichart-Perera, O.; Mier-Cabrera, J.; Flores-Robles, C.M.; Martinez-Cruz, N.; Arce-Sanchez, L.; Alvarado-Maldonado, I.N.; Montoya-Estrada, A.; Romo-Yanez, J.; Rodriguez-Cano, A.M.; Estrada-Gutierrez, G., et al. Intensive medical nutrition therapy alone or with added metformin to prevent gestational diabetes mellitus among high-risk mexican women: a randomized clinical trial. Nutrients 2022, 14, doi:https://doi.org/10.3390/nu14010062. | **Wrong population** |
| Pfieffer, M.L. Polycystic ovary syndrome: Diagnosis and management. Nurse Practitioner 2019, 44, 30-36, doi:10.1097/01.NPR.0000553398.50729.c0. | **Wrong study design** |
| Pkhaladze, L.; Russo, M.; Unfer, V.; Nordio, M.; Basciani, S.; Khomasuridze, A. Treatment of lean PCOS teenagers: a follow-up comparison between Myo-Inositol and oral contraceptives. European review for medical and pharmacological sciences 2021, 25, 7476-7485, doi:https://dx.doi.org/10.26355/eurrev_202112_27447. | **Wrong comparator** |
| Poojari, P.; Padgaonkar, A.; Paramanya, A.; Ali, A. Compendium of polycystic ovarian syndrome and its relevance in glycation and diabetes. Journal of Experimental and Clinical Medicine (Turkey) 2022, 39, 256-268, doi:https://dx.doi.org/10.52142/omujecm.39.1.49. | **Wrong study design** |
| Pourghasem, S.; Bazarganipour, F.; Taghavi, S.A.; Kutenaee, M.A. The effectiveness of inositol and metformin on infertile polycystic ovary syndrome women with resistant to letrozole. Archives of gynecology and obstetrics 2019, https://doi.org/10.1007/s00404-019-05064-5, doi:https://doi.org/10.1007/s00404-019-05064-5. | **Wrong outcome** |
| Powell, A. Choosing the Right Oral Contraceptive Pill for Teens. Pediatric clinics of North America 2017, 64, 343-358, doi:https://dx.doi.org/10.1016/j.pcl.2016.11.005. | **Wrong study design** |
| Practice Committee of the American Society for Reproductive Medicine. Electronic address, A.a.o.; Practice Committee of the American Society for Reproductive, M.; Penzias A, B.K.B.S.C.C.F.T.F.G.G.S.G.C.H.K.L.B.A.M.J.O.R.P. Role of metformin for ovulation induction in infertile patients with polycystic ovary syndrome (PCOS): a guideline. Fertility and sterility 2017, 108, 426-441, doi:https://dx.doi.org/10.1016/j.fertnstert.2017.06.026. | **Wrong comparator** |
| Pradas, I.; Rovira-Llopis, S.; Naudi, A.; Banuls, C.; Rocha, M.; Hernandez-Mijares, A.; Pamplona, R.; Victor, V.M.; Jove, M. Metformin induces lipid changes on sphingolipid species and oxidized lipids in polycystic ovary syndrome women. Scientific reports 2019, 9, 16033, doi:https://dx.doi.org/10.1038/s41598-019-52263-w. | **Wrong study design** |
| Pundir, J.; Psaroudakis, D.; Savnur, P.; Bhide, P.; Sabatini, L.; Teede, H.; Coomarasamy, A.; Khan, K.; Thangaratinam, S. Inositol treatment of anovulation in women with polycystic ovary syndrome: a meta-analysis of randomised trials. Human reproduction (Oxford, England) 2017, 32, i448. | **Wrong intervention** |
| Pundir, J.; Psaroudakis, D.; Savnur, P.; Bhide, P.; Sabatini, L.; Teede, H.; Coomarasamy, A.; Thangaratinam, S. Inositol treatment of anovulation in women with polycystic ovary syndrome: a meta-analysis of randomised trials. BJOG: An International Journal of Obstetrics & Gynaecology 2018, 125, 299-308, doi:10.1111/1471-0528.14754. | **Wrong intervention** |
| Rajasekaran, K.; Malhotra, N. Randomised control trial comparing the effects of myoinositol to metformin on ART outcome in women with PCOS undergoing In-vitro fertilisation (IVF) cycle. Human reproduction. Conference: 36th annual meeting of the european human reproduction and embryology. ESHRE. Virtual meeting 2020, 35 Suppl 1, i396. | **Wrong publication type** |
| Rajasekaran, K.; Malhotra, N.; Mahey, R.; Khadgawat, R.; Kalaivani, M. Myoinositol versus metformin pretreatment in GnRH-antagonist cycle for women with PCOS undergoing IVF: a double-blinded randomized controlled study. Gynecological endocrinology : the official journal of the International Society of Gynecological Endocrinology 2022, 38, 140-147, doi:https://dx.doi.org/10.1080/09513590.2021.1981282. | **Wrong comparator** |
| Rani, N.; Kumar, P.; Mishra, A.; Sankuratri, B.; Sethi, S.; Gelada, K.; Tiwari, H. Efficacy of spironolactone in adult acne in polycystic ovary syndrome patients an original research. Journal of Pharmacy and Bioallied Sciences 2021, 13, S1659-S1663, doi:https://dx.doi.org/10.4103/jpbs.jpbs_391_21. | **Wrong study design** |
| Raperport, C.; Chronopoulou, E.; Homburg, R. Effects of metformin treatment on pregnancy outcomes in patients with polycystic ovary syndrome. Expert review of endocrinology & metabolism 2021, 16, 37-47, doi:https://dx.doi.org/10.1080/17446651.2021.1889366. | **Wrong study design** |
| Rapisarda, A.M.C.; Brescia, R.; Sapia, F.; Valenti, G.; Sarpietro, G.; Di Gregorio, L.M.; Gatta, A.N.D.; La Rosa, V.L.; Sergiampietri, C.; Corte, L.D., et al. Combined oral contraceptive in adolescent and young adult women: Current evidence and future perspectives. Current Women's Health Reviews 2019, 15, 109-118, doi:http://dx.doi.org/10.2174/1573404814666180914162053. | **Wrong study design** |
| Rashid, A.; Ganie, M.A.; Wani, I.A.; Bhat, G.A.; Shaheen, F.; Wani, I.A.; Shrivastava, M.; Shah, Z.A. Differential Impact of Insulin Sensitizers vs. Anti-Androgen on Serum Leptin Levels in Vitamin D Replete PCOS Women: A Six Month Open Labeled Randomized Study. Hormone and metabolic research = Hormon- und Stoffwechselforschung = Hormones et metabolisme 2020, 52, 89-94, doi:https://dx.doi.org/10.1055/a-1084-5441. | **Fulltext not obtainable** |
| Rashid, R.; Mir, S.A.; Kareem, O.; Ali, T.; Ara, R.; Malik, A.; Amin, F.; Bader, G.N. Polycystic ovarian syndrome-current pharmacotherapy and clinical implications. Taiwanese journal of obstetrics & gynecology 2022, 61, 40-50, doi:https://dx.doi.org/10.1016/j.tjog.2021.11.009. | **Wrong study design** |
| Rastegar, F.; Rezaee, Z.; Saedi, N.; Memari, R.; Tajpour, M. Comparison of Effect of Metformin Versus Combination of Folic Acid/Myo-inositol in Infertile Women with Poly Cystic Ovary Syndrome Undergoing in Vitro Fertilization: A Randomized Clinical Trial. Biomedical Research and Therapy 2021, 8, 4734, doi:https://doi.org/10.15419/bmrat.v8i12.710. | **Wrong comparator** |
| Rezk, M.; Shaheen, A.-E.; Saif El-Nasr, I. Clomiphene citrate combined with metformin versus letrozole for induction of ovulation in clomiphene-resistant polycystic ovary syndrome: a randomized clinical trial. Gynecological endocrinology : the official journal of the International Society of Gynecological Endocrinology 2018, 34, 298-300, doi:https://dx.doi.org/10.1080/09513590.2017.1395838. | **Wrong intervention** |
| Rodriguez-Gutierrez, R.; Montes-Villarreal, J.; Rodriguez-Velver, K.V.; Gonzalez-Velazquez, C.; Salcido-Montenegro, A.; Elizondo-Plazas, A.; Gonzalez-Gonzalez, J.G. Metformin Use and Vitamin B12 Deficiency: Untangling the Association. The American journal of the medical sciences 2017, 354, 165-171, doi:https://dx.doi.org/10.1016/j.amjms.2017.04.010. | **Wrong study design** |
| Rogowicz-Frontczak, A.; Majchrzak, A.; Zozulińska-Ziółkiewicz, D. Insulin resistance in endocrine disorders -- treatment options. Polish Journal of Endocrinology / Endokrynologia Polska 2017, 68, 334-350, doi:10.5603/EP.2017.0026.  Romanski, P.; Stanic, A.K. Practical Approach to the PCOS Patient. Current Obstetrics and Gynecology Reports 2017, 6, 11, doi:https://doi.org/10.1007/s13669-017-0190-6. | **Wrong study design** |
| oy, S.B.; Roy, S.B. A Study of the Effect of Metformin Versus Myo-Inositol in the Management of PCOS &mdash; A Randomised Controlled Trial. Journal of the Indian Medical Association 2020, 118, 40. | **Fulltext not obtainable** |
| Ruan, X.; Gu, M.; Mueck, A.O. Pcos patients need anti-androgenic pretreatment before pregnancy. International journal of gynaecology and obstetrics 2018, 143, 809, doi:https://doi.org/10.1002/ijgo.12583. | **Wrong publication type** |
| Ruan, X.; Kubba, A.; Aguilar, A.; Mueck, A.O. Use of cyproterone acetate/ethinylestradiol in polycystic ovary syndrome: rationale and practical aspects. European journal of contraception & reproductive health care 2017, 22, 183-190, doi:10.1080/13625187.2017.1317735. | **Wrong study design** |
| Ruan, X.; Li, M.; Mueck, A.O. Why does Polycystic Ovary Syndrome (PCOS) Need Long-term Management? Current pharmaceutical design 2018, 24, 4685-4692, doi:https://dx.doi.org/10.2174/1381612825666190130104922. | **Wrong study design** |
| Ryssdal, M.; Vanky, E.; Stokkeland, L.M.T.; Jarmund, A.H.; Steinkjer, B.; Lovvik, T.S.; Madssen, T.S.; Iversen, A.C.; Giskeodegard, G.F. Y-012. Metformin changes serum cytokines in pregnant women with polycystic ovary syndrome. Y-012. Metformin changes serum cytokines in pregnant women with polycystic ovary syndrome 2021, 25, e21, doi:https://doi.org/10.1016/j.preghy.2021.07.017. | **Wrong publication type** |
| Sadeeqa, S.; Mustafa, T.; Latif, S. Polycystic ovarian syndrome-related depression in adolescent girls: A Review. Journal of Pharmacy and Bioallied Sciences 2018, 10, 55-59, doi:http://dx.doi.org/10.4103/JPBS.JPBS_1_18. | **Wrong study design** |
| Sadeghpoor, S.; Bolandghamat, B.; Sharajabad, F.A. The possibility and management sterateghies of pregnancy in women with polycystic ovary syndrome: A review article. Journal of Reproduction and Infertility 2017, 18, 231. | **Wrong study design** |
| Salehpour, S.; Nazari, L. New treatment in PCOS. International Journal of Reproductive BioMedicine 2017, 15, 1. | **Wrong study design** |
| Sam, S.; Ehrmann, D.A. Metformin therapy for the reproductive and metabolic consequences of polycystic ovary syndrome. Diabetologia 2017, 60, 1656-1661, doi:https://dx.doi.org/10.1007/s00125-017-4306-3. | **Wrong study design** |
| Sathyapalan, T.; Javed, Z.; Kilpatrick, E.S.; Coady, A.-M.; Atkin, S.L. Endocannabinoid receptor blockade increases vascular endothelial growth factor and inflammatory markers in obese women with polycystic ovary syndrome. Clinical endocrinology 2017, 86, 384-387, doi:https://dx.doi.org/10.1111/cen.13239. | **Wrong comparator** |
| Scheen, A.J.; Philips, J.C.; Kridelka, F. [Role of metformin in gynaecology and obstetrics]. Comment je traite ... Place de la metformine en gynecologie-obstetrique. 2018, 73, 597-602. | **Wrong language** |
| Scioscia, M.; Fascilla, F.; Bettocchi, S. Re: Inositol treatment of anovulation in women with polycystic ovary syndrome: a meta-analysis of randomised trials. | **Wrong publication type** |
| Pundir J, Psaroudakis D, Savnur P, et al. Inositol treatment of anovulation in women with polycystic ovary syndrome: a meta-analysis of randomised trials. BJOG 2018;125:299-308. Wiley-Blackwell: Malden, Massachusetts, 2018; Vol. 125, pp 385-385. | **Wrong intervention** |
| Scioscia, M.; Fascilla, F.; Bettocchi, S. Re: Inositol treatment of anovulation in women with polycystic ovary syndrome: a meta-analysis of randomised trials. BJOG : an international journal of obstetrics and gynaecology 2018, 125, 385, doi:https://dx.doi.org/10.1111/1471-0528.14810. | **Wrong publication type** |
| Seyam, E.; Hefzy, E. Long-term effects of combined simvastatin and metformin treatment on the clinical abnormalities and ovulation dysfunction in single young women with polycystic ovary syndrome. Gynecological endocrinology : the official journal of the International Society of Gynecological Endocrinology 2018, 34, 1073-1080, doi:https://dx.doi.org/10.1080/09513590.2018.1490405. | **Wrong population** |
| Shahnazi, M.; Farshbafkhalili, A.; Ghahremaninasab, P. Comparing the effects of combined low-dose oral contraceptives and vitex agnus on the improvement of symptoms polycystic ovarian syndrome: a triple-blind, randomized, controlled clinical trial. Journal of reproduction and infertility. Conference: 3rd international congress of the iranian society of embryology and reproductive biology, ISERB 2017, 18 Suppl 2, 209. | **Fulltext not obtainable** |
| Shahnazi, M.; Farshbafkhalili, A.; Ghahremaninasab, P. Comparing the effects of combined low-dose oral contraceptives and vitex agnus on the improvement of symptoms polycystic ovarian syndrome: A triple-blind, randomized, controlled clinical trial. Journal of Reproduction and Infertility 2017, 18, 209-210. | **Wrong publication type** |
| Shahriar, S.; Bahrami, S.; Sohran, F. Reviewing the effects of metformin on ovulation of women diagnosed with polycystic ovary syndrome (PCOS). Journal of Reproduction and Infertility 2018, 19, 119-120. | **Wrong study design** |
| Sharma, A.; Welt, C.K. Practical Approach to Hyperandrogenism in Women. The Medical clinics of North America 2021, 105, 1099-1116, doi:https://dx.doi.org/10.1016/j.mcna.2021.06.008. | **Wrong study design** |
| Sharma, S.; Mathur, D.K.; Paliwal, V.; Bhargava, P. Efficacy of Metformin in the Treatment of Acne in Women with Polycystic Ovarian Syndrome: A Newer Approach to Acne Therapy. Journal of Clinical & Aesthetic Dermatology 2019, 12, 34-38. | **Wrong study design** |
| Shen, W.; Jin, B.; Han, Y.; Wang, H.; Jiang, H.; Zhu, L.; Han, M.; Zhang, J.; Zhang, Y. The Effects of Salvia miltiorrhiza on Reproduction and Metabolism in Women with Polycystic Ovary Syndrome: A Systematic Review and Meta-Analysis. Evidence-based Complementary & Alternative Medicine (eCAM) 2021, 10.1155/2021/9971403, 1-12, doi:10.1155/2021/9971403. | **Wrong intervention** |
| Shokrpour, M.; Foroozanfard, F.; Afshar Ebrahimi, F.; Vahedpoor, Z.; Aghadavod, E.; Ghaderi, A.; Asemi, Z. Comparison of myo-inositol and metformin on glycemic control, lipid profiles, and gene expression related to insulin and lipid metabolism in women with polycystic ovary syndrome: a randomized controlled clinical trial. Gynecological Endocrinology 2019, 35, 406, doi:https://doi.org/10.1080/09513590.2018.1540570. | **Wrong comparator** |
| Showell, M.G.; Mackenzie-Proctor, R.; Jordan, V.; Hodgson, R.; Farquhar, C. Inositol for subfertile women with polycystic ovary syndrome. The Cochrane database of systematic reviews 2018, 12, CD012378, doi:https://dx.doi.org/10.1002/14651858.CD012378.pub2. | **Wrong outcome** |
| Shuai, W.; Tang, Z.; Gu, W.; Tong, X.; Cao, J. Impact of metformin on low-grade chronic inflammatory mediators in women with polycystic ovary syndrome: A meta-analysis. Latin American Journal of Pharmacy 2020, 39, 1388-1399. | **Fulltext not obtainable** |
| Siamashvili, M.; Davis, S.N. Update on the effects of GLP-1 receptor agonists for the treatment of polycystic ovary syndrome. Expert review of clinical pharmacology 2021, 14, 1081-1089, doi:https://dx.doi.org/10.1080/17512433.2021.1933433. | **Wrong study design** |
| Silva-Bermudez, L.S.; Toloza, F.J.K.; Perez-Matos, M.C.; de Souza, R.J.; Banfield, L.; Vargas-Villanueva, A.; Mendivil, C.O. Effects of oral contraceptives on metabolic parameters in adult premenopausal women: A meta-analysis. Endocrine Connections 2020, 9, 978-998, doi:http://dx.doi.org/10.1530/EC-20-0423. | **Wrong comparator** |
| Sohrevardi, S.M.; Heydari, B.; Azarpazhooh, M.R.; Teymourzadeh, M.; Simental-Mendia, L.E.; Atkin, S.L.; Sahebkar, A.; Karimi-Zarchi, M. Therapeutic Effect of Curcumin in Women with Polycystic Ovary Syndrome Receiving Metformin: A Randomized Controlled Trial. Advances in experimental medicine and biology 2021, 1308, 109-117, doi:https://dx.doi.org/10.1007/978-3-030-64872-5_9. | **Wrong comparator** |
| Soldat-Stankovic, V.; Pejicic, S.P.; Stankovic, S.; Jovanic, J.; Bjekic-Macut, J.; Livadas, S.; Ognjanovic, S.; Mastorakos, G.; Micic, D.; Macut, D. THE EFFECT OF MYOINOSITOL AND METFORMIN ON CARDIOVASCULAR RISK FACTORS IN WOMEN WITH POLYCYSTIC OVARY SYNDROME: a RANDOMIZED CONTROLLED TRIAL. Acta endocrinologica 2021, 17, 241, doi:https://doi.org/10.4183/aeb.2021.241. | **Wrong comparator** |
| Soldat-Stankovic, V.; Popovic-Pejicic, S.; Stankovic, S.; Prtina, A.; Malesevic, G.; Bjekic-Macut, J.; Livadas, S.; Ognjanovic, S.; Mastorakos, G.; Micic, D., et al. The effect of metformin and myoinositol on metabolic outcomes in women with polycystic ovary syndrome: role of body mass and adiponectin in a randomized controlled trial. Journal of endocrinological investigation 2022, 45, 583-595, doi:https://dx.doi.org/10.1007/s40618-021-01691-5. | **Wrong comparator** |
| Soliman, A.; De Sanctis, V.; Alaaraj, N.; Hamed, N. The clinical application of metformin in children and adolescents: A short update. Acta bio-medica : Atenei Parmensis 2020, 91, e2020086, doi:https://dx.doi.org/10.23750/abm.v91i3.10127. | **Wrong study design** |
| Song, S.Y.; Yang, J.B.; Song, M.S.; Oh, H.Y.; Lee, G.W.; Lee, M.; Ko, Y.B.; Lee, K.H.; Chang, H.K.; Kwak, S.M., et al. Effect of pretreatment with combined oral contraceptives on outcomes of assisted reproductive technology for women with polycystic ovary syndrome: a meta-analysis. Archives of Gynecology & Obstetrics 2019, 300, 737-750, doi:10.1007/s00404-019-05210-z. | **Wrong outcome** |
| Song, Y.; Wang, H.; Huang, H.; Zhu, Z. Comparison of the efficacy between NAC and metformin in treating PCOS patients: a meta-analysis. Gynecological endocrinology : the official journal of the International Society of Gynecological Endocrinology 2020, 36, 204-210, doi:https://dx.doi.org/10.1080/09513590.2019.1689553. | **Wrong comparator** |
| Song, Y.; Wang, H.; Zhu, Z.; Huang, H. Effects of Metformin and Exercise in Polycystic Ovary Syndrome: Systematic Review and Meta-Analysis. Hormone and metabolic research = Hormon- und Stoffwechselforschung = Hormones et metabolisme 2021, 53, 738-745, doi:https://dx.doi.org/10.1055/a-1666-8979. | **Fulltext not obtainable** |
| Sova, H.; Unkila-Kallio, L.; Tiitinen, A.; Hippelainen, M.; Perheentupa, A.; Tinkanen, H.; Puukka, K.; Bloigu, R.; Piltonen, T.; Tapanainen, J., et al. Decrease in serum AMH levels during prepregnancy metformin therapy associates with improved pregnancy and live-birth rates in women with PCOS: a multicentre, double-blind, placebo-controlled RCT. Human reproduction (Oxford, England) 2019, 34, i145. | **Wrong publication type** |
| Sova, H.; Unkila-Kallio, L.; Tiitinen, A.; Hippelainen, M.; Perheentupa, A.; Tinkanen, H.; Puukka, K.; Bloigu, R.; Piltonen, T.; Tapanainen, J., et al. Decrease in serum AMH levels during prepregnancy metformin therapy associates with improved pregnancy and live-birth rates in women with PCOS: a multicentre, double-blind, placebo-controlled RCT. Human reproduction. Conference: 35th annual meeting of the european society of human reproduction and embryology. ESHRE. Vienna, austria 2019, 34 Suppl 1. | **Wrong publication type** |
| Stefanaki, C.; Bacopoulou, F.; Kandaraki, E.; Boschiero, D.; Diamandi-Kandarakis, E. Lean Women on Metformin and Oral Contraceptives for Polycystic Ovary Syndrome Demonstrate a Dehydrated Osteosarcopenic Phenotype: A Pilot Study. Nutrients 2019, 11, 2055, doi:10.3390/nu11092055. | **Wrong study design** |
| Stener-Victorin, E.; Zhang, H.; Li, R.; Friden, C.; Li, D.; Wang, W.; Wang, H.; Chang, C.; Li, S.; Huo, Z., et al. Acupuncture or metformin to improve insulin resistance in women with polycystic ovary syndrome: study protocol of a combined multinational cross sectional case-control study and a randomised controlled trial. BMJ open 2019, 9, e024733, doi:https://dx.doi.org/10.1136/bmjopen-2018-024733. | **Wrong study design** |
| Stewart, C.E.; Sohrabji, F.; Agarwal, A. Gonadal hormones and stroke risk: PCOS as a case study. Frontiers in Neuroendocrinology 2020, 58, doi:https://dx.doi.org/10.1016/j.yfrne.2020.100853. | **Wrong study design** |
| Street, M.E.; Cirillo, F.; Catellani, C.; Dauriz, M.; Lazzeroni, P.; Sartori, C.; Moghetti, P. Current treatment for polycystic ovary syndrome: focus on adolescence. Minerva pediatrica 2020, 72, 288-311, doi:https://dx.doi.org/10.23736/S0026-4946.20.05861-2. | **Wrong study design** |
| Stridsklev, S.; Salvesen, O.; Salvesen, K.A.; Carlsen, S.M.; Husoy, M.A.; Vanky, E. Uterine artery doppler in pregnancy: women with PCOS compared to healthy controls. Endocrine reviews 2017, 38. | **Wrong publication type** |
| Sung, C.T.; Chao, T.; Lee, A.; Foulad, D.P.; Choi, F.; Juhasz, M.; Dobry, A.; Mesinkovska, N.A. Oral Metformin for Treating Dermatological Diseases: A Systematic Review. Journal of drugs in dermatology : JDD 2020, 19, 713-720, doi:https://dx.doi.org/10.36849/JDD.2020.4874. | **Wrong comparator** |
| Syed, S.Z.; Akram, F.; Aftab Hassan, S.M. Comparison of efficacy of metformin versus Pioglitazone on ovulation in patients of polycystic ovarian syndrome. Pakistan Journal of Medical and Health Sciences 2018, 12, 1240-1242. | **Wrong outcome** |
| Tagliaferri, V.; Romualdi, D.; Immediata, V.; De Cicco, S.; Di Florio, C.; Lanzone, A.; Guido, M. Metformin vs myoinositol: which is better in obese polycystic ovary syndrome patients? A randomized controlled crossover study. Clinical endocrinology 2017, 86, 725-730, doi:https://dx.doi.org/10.1111/cen.13304. | **Wrong comparator** |
| Talaat, B.; Ammar, I.M.M. The added value of cinnamon to metformin in controlling symptoms of polycystic ovary syndrome, a randomized controlled trial. Middle East Fertility Society Journal 2018, 23, 440-445, doi:http://dx.doi.org/10.1016/j.mefs.2018.03.005. | **Wrong comparator** |
| Tan, J.; Zhou, G.J.; Wang, Q.Y.; Liu, T.T.; Cao, Q.; Huang, W. [Effect of metformin and rosiglitazone in non-obese polycystic ovary syndrome women with insulin resistance]. Zhonghua fu chan ke za zhi 2021, 56, 467-473, doi:https://dx.doi.org/10.3760/cma.j.cn112141-20210424-00224. | **Wrong language** |
| Tang, L.; Ye, J.; Shi, Y.; Zhu, X. Association between CD16++ monocytes in peripheral blood and clinical features and short-term therapeutic effects of polycystic ovary syndrome. International Journal of Gynecology & Obstetrics 2019, 145, 12-17, doi:10.1002/ijgo.12779. | **Wrong study design** |
| Tao, T.; Wu, P.; Wang, Y.; Liu, W. Comparison of glycemic control and β-cell function in new onset T2DM patients with PCOS of metformin and saxagliptin monotherapy or combination treatment. BMC endocrine disorders 2018, 18, 1-1, doi:10.1186/s12902-018-0243-5. | **Wrong population** |
| Tauqir, S.; Israr, M.; Rauf, B.; Malik, M.O.; Habib, S.H.; Shah, F.A.; Usman, M.; Raza, M.A.; Shah, I.; Badshah, H., et al. Acetyl-L-Carnitine Ameliorates Metabolic and Endocrine Alterations in Women with PCOS: A Double-Blind Randomized Clinical Trial. Advances in therapy 2021, 38, 3842-3856, doi:https://dx.doi.org/10.1007/s12325-021-01789-5. | **Wrong comparator** |
| Tay, C.T.; Joham, A.E.; Hiam, D.S.; Gadalla, M.A.; Pundir, J.; Thangaratinam, S.; Teede, H.J.; Moran, L.J. Pharmacological and surgical treatment of nonreproductive outcomes in polycystic ovary syndrome: An overview of systematic reviews. Clinical endocrinology 2018, 89, 535-553, doi:https://dx.doi.org/10.1111/cen.13753. | **Wrong study design** |
| Tehrani, F.R.; Amiri, M. Polycystic ovary syndrome in adolescents: Challenges in diagnosis and treatment. International Journal of Endocrinology and Metabolism 2019, 17, e91554, doi:http://dx.doi.org/10.5812/ijem.91554. | **Wrong study design** |
| Tennilä, J.; Jääskeläinen, J.; Utriainen, P.; Voutilainen, R.; Häkkinen, M.; Auriola, S.; Morin-Papunen, L.; Liimatta, J. PCOS Features and Steroid Profiles Among Young Adult Women with a History of Premature Adrenarche. Journal of Clinical Endocrinology & Metabolism 2021, 106, e3335-e3345, doi:10.1210/clinem/dgab385. | **Wrong study design** |
| Toosy, S.; Sodi, R.; Pappachan, J.M. Lean polycystic ovary syndrome (PCOS): an evidence-based practical approach. Journal of Diabetes and Metabolic Disorders 2018, 17, 277-285, doi:http://dx.doi.org/10.1007/s40200-018-0371-5. | **Wrong study design** |
| Trouva, A.; Alvarsson, M.; Calissendorff, J.; Asvold, B.O.; Vanky, E.; Hirschberg, A.L. Thyroid Status During Pregnancy in Women With Polycystic Ovary Syndrome and the Effect of Metformin. Frontiers in endocrinology 2022, 13, 772801, doi:https://dx.doi.org/10.3389/fendo.2022.772801. | **Wrong outcome** |
| Tso, L.O.; Costello, M.F.; Albuquerque, L.E.T.; Andriolo, R.B.; Macedo, C.R. Metformin treatment before and during IVF or ICSI in women with polycystic ovary syndrome. The Cochrane database of systematic reviews 2020, 12, CD006105, doi:https://dx.doi.org/10.1002/14651858.CD006105.pub4. | **Wrong population** |
| Tzotzas, T.; Karras, S.N.; Katsiki, N. Glucagon-Like Peptide-1 (GLP-1) Receptor Agonists in the Treatment of Obese Women with Polycystic Ovary Syndrome. Current vascular pharmacology 2017, 15, 218-229, doi:https://dx.doi.org/10.2174/1570161114666161221115324. | **Wrong study design** |
| Udesen, P.B.; Glintborg, D.; Sorensen, A.E.; Svendsen, R.; Nielsen, N.L.S.; Wissing, M.L.M.; Andersen, M.S.; Englund, A.L.M.; Dalgaard, L.T. Metformin decreases mir-122, mir-223 and mir-29a in women with polycystic ovary syndrome. Endocrine Connections 2020, 9, 1075, doi:https://doi.org/10.1530/EC-20-0195. | **Wrong outcome** |
| Underdal, M.O.; Salvesen, Ø.; Henriksen, A.H.; Andersen, M.; Vanky, E. Impaired Respiratory Function in Women With PCOS Compared With Matched Controls From a Population-Based Study. Journal of Clinical Endocrinology & Metabolism 2019, 10.1210/clinem/dgz053, N.PAG-N.PAG, doi:10.1210/clinem/dgz053. | **Wrong study design** |
| Underdal, M.O.; Stridsklev, S.; Andresen, M.S.; Vanky, E. Metabolic health in women with PCOS-5-11 years' followup after metformin or placebo in pregnancy. Endocrine reviews 2017, 38. | **Fulltext not obtainable** |
| Underdal, M.O.; Stridsklev, S.; Oppen, I.H.; Høgetveit, K.; Andersen, M.S.; Vanky, E. Does Metformin Treatment During Pregnancy Modify the Future Metabolic Profile in Women With PCOS? Journal of Clinical Endocrinology & Metabolism 2018, 10.1210/jc.2018-00485, N.PAG-N.PAG, doi:10.1210/jc.2018-00485. | **Wrong population** |
| Vatopoulou, A.; Tziomalos, K. Management of obesity in adolescents with polycystic ovary syndrome. Expert opinion on pharmacotherapy 2020, 21, 207-211, doi:https://dx.doi.org/10.1080/14656566.2019.1701655. | **Wrong study design** |
| Vedtofte, L.; Foghsgaard, S.; Zierau, L.; VilsbØLl, T.; Knop, F.K. 1186-P: Lean Women with Polycystic Ovary Syndrome and Insulin Resistance Have Normal Incretin Effect, which Is Unaffected by Metformin Therapy. Diabetes 2019, 68, N.PAG-N.PAG, doi:10.2337/db19-1186-P. | **Wrong publication type** |
| Venter, A. Obesity, Oligomenorrhoea and PCOS in Adolescence. Obstetrics and Gynaecology Forum 2018, 28, 27-30. | **Wrong study design** |
| Vine, D.; Proctor, E.; Weaver, O.; Ghosh, M.; Maximova, K.; Proctor, S. A Pilot Trial: fish Oil and Metformin Effects on ApoB-Remnants and Triglycerides in Women with Polycystic Ovary Syndrome. Journal of the Endocrine Society 2021, 5, doi:https://doi.org/10.1210/jendso/bvab114. | **Wrong comparator** |
| Walker, K.; Decherney, A.H.; Saunders, R. Menstrual Dysfunction in PCOS. Clinical Obstetrics & Gynecology 2021, 64, 119-125, doi:10.1097/GRF.0000000000000596. | **Wrong study design** |
| Wang, J.; Zhu, L.; Hu, K.; Tang, Y.; Zeng, X.; Liu, J.; Xu, J. Effects of metformin treatment on serum levels of C-reactive protein and interleukin-6 in women with polycystic ovary syndrome: a meta-analysis: A PRISMA-compliant article. Medicine 2017, 96, e8183-e8183, doi:10.1097/MD.0000000000008183. | **Wrong comparator** |
| Wang, L.; Liang, R.; Tang, Q.; Zhu, L. An Overview of Systematic Reviews of Using Chinese Medicine to Treat Polycystic Ovary Syndrome. Evidence-based Complementary and Alternative Medicine 2021, 2021, 9935536, doi:http://dx.doi.org/10.1155/2021/9935536 | **Wrong intervention** |
| Wang, R.; Kim, B.V.; van Wely, M.; Johnson, N.P.; Costello, M.F.; Zhang, H.; Ng, E.H.Y.; Legro, R.S.; Bhattacharya, S.; Norman, R.J., et al. Treatment strategies for women with WHO group II anovulation: systematic review and network meta-analysis. BMJ (Clinical research ed.) 2017, 356, j138, doi:https://dx.doi.org/10.1136/bmj.j138. | **Wrong population** |
| Wang, R.; Li, W.; Bordewijk, E.M.; Legro, R.S.; Zhang, H.; Wu, X.; Gao, J.; Morin-Papunen, L.; Homburg, R.; Konig, T.E., et al. First-line ovulation induction for polycystic ovary syndrome: an individual participant data meta-analysis. Human reproduction update 2019, 25, 717-732, doi:https://dx.doi.org/10.1093/humupd/dmz029. | **Wrong outcome** |
| Wang, Y.-W.; He, S.-J.; Feng, X.; Cheng, J.; Luo, Y.-T.; Tian, L.; Huang, Q. Metformin: a review of its potential indications. Drug design, development and therapy 2017, 11, 2421-2429, doi:https://dx.doi.org/10.2147/DDDT.S141675. | **Wrong study design** |
| Wawrzkiewicz-Jalowiecka, A.; Kowalczyk, K.; Trybek, P.; Jarosz, T.; Radosz, P.; Setlak, M.; Madej, P. In Search of New Therapeutics-Molecular Aspects of the PCOS Pathophysiology: Genetics, Hormones, Metabolism and Beyond. International journal of molecular sciences 2020, 21, doi:https://dx.doi.org/10.3390/ijms21197054. | **Wrong study design** |
| Wen, Y.; Ma, H.L.; Wu, X.K. Acupuncture and clomiphene interventions in PCOS conversely affect the insulin resistance profiles in early pregnancy subjects: a secondary analysis of a randomized controlled trial. Journal of obstetrics and gynaecology research 2017, 43, 160, doi:https://doi.org/10.1111/jog.13394. | **Wrong publication type** |
| Wenjing, L.; Hongbo, H.; Guofang, Z.; Zhanzhong, M.; Jing, L.; Fanxiang, L.; Li, W.; Hu, H.; Zou, G.; Ma, Z., et al. Therapeutic effects of puerarin on polycystic ovary syndrome: A randomized trial in Chinese women. Medicine 2021, 100, 1-8, doi:10.1097/MD.0000000000026049. | **Wrong comparator** |
| Witchel, S.F.; Oberfield, S.E.; Peña, A.S. Polycystic Ovary Syndrome: Pathophysiology, Presentation, and Treatment With Emphasis on Adolescent Girls. Journal of the Endocrine Society 2019, 3, 1545-1573, doi:10.1210/js.2019-00078. | **Wrong study design** |
| Wiweko, B.; Susanto, C. The Effect of Metformin and Cinnamon on Serum Anti-Mullerian Hormone in Women Having PCOS: a Double-Blind, Randomized, Controlled Trial. Journal of Human Reproductive Sciences 2017, 10, 31, doi:https://doi.org/10.4103/jhrs.JHRS9016. | **Wrong comparator** |
| Wojciechowska, A.; Osowski, A.; Jozwik, M.; Gorecki, R.; Rynkiewicz, A.; Wojtkiewicz, J. Inositols' Importance in the Improvement of the Endocrine-Metabolic Profile in PCOS. International journal of molecular sciences 2019, 20, doi:https://dx.doi.org/10.3390/ijms20225787. | **Wrong study design** |
| Woodward, A.; Broom, D.; Harrop, D.; Lahart, I.; Carter, A.; Dalton, C.; Metwally, M.; Klonizakis, M. The effects of physical exercise on cardiometabolic outcomes in women with polycystic ovary syndrome not taking the oral contraceptive pill: a systematic review and meta-analysis. Journal of Diabetes and Metabolic Disorders 2019, 18, 597-612, doi:http://dx.doi.org/10.1007/s40200-019-00425-y. | **Wrong intervention** |
| Wu, Y.; Tu, M.; Huang, Y.; Liu, Y.; Zhang, D. Association of Metformin With Pregnancy Outcomes in Women With Polycystic Ovarian Syndrome Undergoing In Vitro Fertilization: A Systematic Review and Meta-analysis. JAMA network open 2020, 3, e2011995-e2011995, doi:10.1001/jamanetworkopen.2020.11995. | **Wrong outcome** |
| Xie, L.; Zhang, D.; Ma, H.; He, H.; Xia, Q.; Shen, W.; Chang, H.; Deng, Y.; Wu, Q.; Cong, J., et al. The Effect of Berberine on Reproduction and Metabolism in Women with Polycystic Ovary Syndrome: A Systematic Review and Meta-Analysis of Randomized Control Trials. Evidence-based Complementary & Alternative Medicine (eCAM) 2019, 10.1155/2019/7918631, 1-15, doi:10.1155/2019/7918631. | **Wrong intervention** |
| Xu, J.; Zuo, Y. [Efficacy of acupuncture as adjunctive treatment on infertility patients with polycystic ovary syndrome]. Zhongguo zhen jiu = Chinese acupuncture & moxibustion 2018, 38, 358-361, doi:https://dx.doi.org/10.13703/j.0255-2930.2018.04.004. | **Wrong language** |
| Xu, Q.; Xie, Q. Long-term effects of prenatal exposure to metformin on the health of children based on follow-up studies of randomized controlled trials: a systematic review and meta-analysis. Springer Nature: , <Blank>, 2019; Vol. 299, pp 1295-1303. | **Wrong outcome** |
| Xu, Z.; Meng, L.; Pan, C.; Chen, X.; Huang, X.; Yang, H. Does oral contraceptives pretreatment affect the pregnancy outcome in polycystic ovary syndrome women undergoing ART with GnRH agonist protocol? Gynecological endocrinology : the official journal of the International Society of Gynecological Endocrinology 2019, 35, 124-127, doi:https://dx.doi.org/10.1080/09513590.2018.1500535. | **Wrong study design** |
| Yanbo, L.; Yupei, S.; Jiping, X.; Linlin, C.; Guang, Z.; Liu, Y.; Shao, Y.; Xie, J.; Chen, L.; Zhu, G. The efficacy and safety of metformin combined with simvastatin in the treatment of polycystic ovary syndrome: A meta-analysis and systematic review. Medicine 2021, 100, 1-8, doi:10.1097/MD.0000000000026622. | **Wrong intervention** |
| Yang, D.; Zhao, M.; Tan, J. [Effect of polycystic ovary syndrome treated with the periodic therapy of acupuncture]. Zhongguo zhen jiu = Chinese acupuncture & moxibustion 2017, 37, 825-829, doi:https://dx.doi.org/10.13703/j.0255-2930.2017.08.007. | **Wrong language** |
| Yang, J.; Liu, Y.; Huang, J.; Xu, J.; You, X.; Lin, Q.; Zhang, J.; Dun, J.; Huang, S. [Acupuncture and Chinese medicine of artificial cycle therapy for insulin resistance of polycystic ovary syndrome with phlegm damp type and its mechanism]. Zhongguo zhen jiu = Chinese acupuncture & moxibustion 2017, 37, 1163-1168, doi:https://dx.doi.org/10.13703/j.0255-2930.2017.11.007. | **Wrong language** |
| Yao, K.; Bian, C.; Zhao, X. Association of polycystic ovary syndrome with metabolic syndrome and gestational diabetes: Aggravated complication of pregnancy (Review). Experimental and Therapeutic Medicine 2017, 14, 1271-1276, doi:http://dx.doi.org/10.3892/etm.2017.4642. | **Wrong study design** |
| Yen, H.; Chang, Y.-T.; Yee, F.-J.; Huang, Y.-C. Metformin Therapy for Acne in Patients with Polycystic Ovary Syndrome: A Systematic Review and Meta-analysis. American journal of clinical dermatology 2021, 22, 11-23, doi:10.1007/s40257-020-00565-5. | **Wrong outcome** |
| Young, C.C.; Monge, M. Polycystic Ovary Syndrome in Primary Care: It Takes a Village. Journal for Nurse Practitioners 2019, 15, 694-695, doi:10.1016/j.nurpra.2019.05.008. | **Wrong study design** |
| Yousuf, S.D.; Ganie, M.A.; Jeelani, S.; Mudassar, S.; Shah, Z.A.; Zargar, M.A.; Amin, S.; Wani, I.A.; Rashid, F. Effect of six-month use of oral contraceptive pills on plasminogen activator inhibitor-1 & factor VIII among women with polycystic ovary syndrome: An observational pilot study. The Indian journal of medical research 2018, 148, S151-S155, doi:https://dx.doi.org/10.4103/ijmr.IJMR_1899_17. | **Wrong study design** |
| Zeng, L.; Yang, K. Effectiveness of myoinositol for polycystic ovary syndrome: a systematic review and meta-analysis. Endocrine 2018, 59, 30-38, doi:https://dx.doi.org/10.1007/s12020-017-1442-y. | **Wrong intervention** |
| Zhang, J.; Si, Q.; Li, J. Therapeutic effects of metformin and clomiphene in combination with lifestyle intervention on infertility in women with obese polycystic ovary syndrome. Pakistan journal of medical sciences 2017, 33, 8, doi:https://doi.org/10.12669/pjms.331.11764. | **Wrong intervention** |
| Zhang, J.; Su, M.; Xu, L.; Yang, Z.; Yin, W.; Nie, Y.; Qiao, X.; Cheng, R.; Ma, Y. [Efficacy and metabolic safety of long-term treatment with ethinyl oestradiol/cyproterone and desogestrel/ethinyl oestradiol tablets in women with polycystic ovary syndrome]. Nan fang yi ke da xue xue bao = Journal of Southern Medical University 2018, 38, 917-922, doi:https://dx.doi.org/10.3969/j.issn.1673-4254.2018.08.03. | **Wrong language** |
| Zhang, S.-W.; Zhou, J.; Gober, H.-J.; Leung, W.T.; Wang, L. Effect and mechanism of berberine against polycystic ovary syndrome. Biomedicine & pharmacotherapy = Biomedecine & pharmacotherapie 2021, 138, 111468, doi:https://dx.doi.org/10.1016/j.biopha.2021.111468. | **Wrong study design** |
| Zhang, Y.; Guo, X.; Ma, S.; Ma, H.; Li, H.; Wang, Y.; Qin, Z.; Wu, X.; Han, Y.; Han, Y. The Treatment with Complementary and Alternative Traditional Chinese Medicine for Menstrual Disorders with Polycystic Ovary Syndrome. Evidence-based Complementary & Alternative Medicine (eCAM) 2021, 10.1155/2021/6678398, 1-19, doi:10.1155/2021/6678398. | **Wrong study design** |
| Zhao, J.; Liu, X.; Zhang, W. The Effect of Metformin Therapy for Preventing Gestational Diabetes Mellitus in Women with Polycystic Ovary Syndrome: A Meta-Analysis. Experimental and clinical endocrinology & diabetes : official journal, German Society of Endocrinology [and] German Diabetes Association 2020, 128, 199-205, doi:https://dx.doi.org/10.1055/a-0603-3394. | **Fulltext not obtainable** |
| Zhao, Y.X.; Wang, L.J.; Gong, F.Y.; Pan, H.; Miao, H.; Duan, L.; Yang, H.B.; Zhu, H.J. [Effects of orlistat and metformin on metabolism and gonadal function in overweight or obese patients with polycystic ovary syndrome]. Zhonghua nei ke za zhi 2021, 60, 1165-1168, doi:https://dx.doi.org/10.3760/cma.j.cn112138-20210302-00171. | **Wrong language** |
| Zhou, K.; Zhang, J.; Xu, L.; Lim, C.E.D. Chinese herbal medicine for subfertile women with polycystic ovarian syndrome. The Cochrane database of systematic reviews 2021, 6, CD007535, doi:https://dx.doi.org/10.1002/14651858.CD007535.pub4. | **Wrong intervention** |
| Zimmerman, L.D.; Setton, R.; Pereira, N.; Rosenwaks, Z. Contemporary Management of Polycystic Ovarian Syndrome. Clinical obstetrics and gynecology 2019, 62, 271-281, doi:https://dx.doi.org/10.1097/GRF.0000000000000449. | **Wrong study design** |
